# Transcriptomics in idiopathic pulmonary fibrosis unveiled: a new perspective from differentially expressed genes to therapeutic targets

**DOI:** 10.3389/fimmu.2024.1375171

**Published:** 2024-03-19

**Authors:** Wenzhong Hu, Yun Xu

**Affiliations:** ^1^ Guang’anmen Hospital South Campus, China Academy of Chinese Medical Sciences, Beijing, China; ^2^ People's Hospital of Beijing Daxing District, Capital Medical University, Beijing, China

**Keywords:** idiopathic pulmonary fibrosis, differentially expressed genes, microarray data, eQTL analysis, Mendelian randomization, immune cell infiltration

## Abstract

**Background:**

The underlying molecular pathways of idiopathic pulmonary fibrosis (IPF), a progressive lung condition with a high death rate, are still mostly unknown. By using microarray datasets, this study aims to identify new genetic targets for IPF and provide light on the genetic factors that contribute to the development of IPF.

**Method:**

We conducted a comprehensive analysis of three independent IPF datasets from the Gene Expression Omnibus (GEO) database, employing R software for data handling and normalization. Our evaluation of the relationships between differentially expressed genes (DEGs) and IPF included differential expression analysis, expression quantitative trait loci (eQTL) analysis, and Mendelian Randomization(MR) analyses. Additionally, we used Gene Set Enrichment Analysis (GSEA) and Gene Ontology (GO)/Kyoto Encyclopedia of Genes and Genomes (KEGG) enrichment analysis to explore the functional roles and pathways of these genes. Finally, we validated the results obtained for the target genes.

**Results:**

We identified 486 highly expressed genes and 468 lowly expressed genes that play important roles in IPF. MR analysis identified six significantly co-expressed genes associated with IPF, specifically C12orf75, SPP1, ZG16B, LIN7A, PPP1R14A, and TLR2. These genes participate in essential biological processes and pathways, including macrophage activation and neural system regulation. Additionally, CIBERSORT analysis indicated a unique immune cell distribution in IPF, emphasized the significance of immunological processes in the disease. The MR analysis was consistent with the results of the analysis of variance in the validation cohort, which strengthens the reliability of our MR findings.

**Conclusion:**

Our findings provide new insights into the molecular basis of IPF and highlight the promise of therapeutic interventions. They emphasize the potential of targeting specific molecular pathways for the treatment of IPF, laying the foundation for further research and clinical work.

## 1 Introduction

Idiopathic pulmonary fibrosis (IPF) is a chronic, progressively worsening lung disease characterized by increased fibrosis and deterioration of lung function. This deterioration eventually leads to respiratory failure and death. The disease has a grim prognosis, with patients typically surviving only 3-5 years following diagnosis (1).Therefore, there is an urgent need for new effective treatment alternatives. Despite the increasing incidence and prevalence of IPF, knowledge of the disease remains limited, which poses a significant challenge for the treatment of IPF.

In recent years, IPF research has primarily focused on several aspects, notably the emerging cellular and molecular determinants, growth factors, cytokine pathways, genetic susceptibility, cellular senescence, and the potential of anti-aging drugs in enhancing alveolar epithelial cell function to alleviate pulmonary fibrosis (2–5). In addition, recent studies have found that inhibition of the MARCKS-PIP3 pathway delay the evolution of fibrosis and reduce fibroblast activation (6).

The relationship between inflammation and IPF remains a controversial topic. Although inflammation is implicated in the pathogenesis of the disease, the efficacy of anti-inflammatory drugs remains controversial, as highlighted by the unfavorable results of various multicenter clinical trials (7). IPF is primarily a fibrotic condition driven by abnormal activation of alveolar epithelial cells. This activation triggers the formation of scar tissue, resulting in the destruction of lung structure (8). The advent of antifibrotic drugs such as nintedanib and pirfenidone, which have significantly slowed the progression of IPF, has been a major breakthrough. However, the long-term survival benefits of these drugs remain to be finalized (9). Biologic agents might be a more favorable choice for current idiopathic pulmonary fibrosis treatment approaches. Research indicates that, although the efficacy of most biologics in treating IPF is limited, certain therapeutic strategies have demonstrated potential in improving patient quality of life. Further research is required to confirm the safety and effectiveness of these biologics, providing a new direction for future IPF treatment research (10). This research is dedicated to exploring the intricate mechanisms underlying IPF, with a particular focus on cellular and molecular aspects that could unveil novel therapeutic targets. In light of the limited existing treatments and the high mortality associated with IPF, investigating these novel mechanisms is essential for devising more effective treatment approaches.

This study is dedicated to exploring the complex mechanisms of IPF, with a particular focus on cellular and molecular aspects to reveal new therapeutic targets. Given the limited number of available treatments and the high mortality rate associated with IPF, investigating these novel mechanisms is essential to design more effective therapies.

The main objective of this study was to identify differentially expressed genes (DEGs) in IPF compared to normal samples by analyzing microarray datasets. The study aims to assess the association and causality of these genes with the pathogenesis of IPF through expression quantitative trait loci (eQTL) and MR analysis. In addition, the study will employ Gene Ontology (GO)/Kyoto Encyclopedia of Genomes (KEGG) enrichment analysis and Genome Enrichment Analysis (GSEA) to investigate the potential functional pathways and pathogenesis associated with these DEGs. The study hopes that these approaches will reveal the molecular basis of IPF and lay the foundation for new therapeutic strategies. The study will also suggest areas for future research, emphasizing the importance of a comprehensive understanding of the complex pathobiology of IPF for the development of innovative therapeutic approaches.

## 2 Materials and methods

### 2.1 Data collection

Gene expression datasets and clinical phenotype data matching the search terms “idiopathic pulmonary fibrosis”, “Homo sapiens” and “gene expression” were obtained by microarray dataset analysis. All measured gene expression data and corresponding platform probe annotations are downloadable from the Gene Expression Omnibus (GEO) database (https://www.ncbi.nlm.nih.gov/geo/). For idiopathic pulmonary fibrosis, dataset filtering criteria included a minimum of eight samples, at least four cases and four controls, samples not chemically treated or genetically modified, and the availability of original data or array gene expression profile analysis in the GEO database.

### 2.2 Identification of DEGs

R software (version 4.3.2) was used to read and preprocess datasets GSE24206, GSE53845, and GSE195770 for individual dataset correction. The datasets were then merged, and batch correction and differential analysis were performed on 18 normal samples and 61 idiopathic pulmonary fibrosis samples. The “limma” package was used for classical Bayesian data analysis to filter DEGs, with significance criteria set at P < 0.05 and logFoldChange (LogFC) > 0.585. The “pheatmap” package generated volcano plots and heatmaps of DEGs. Gene expression matrices and annotation files downloaded from the GEO database were used for data normalization and standardization. Principal component analysis (PCA) using the “prcomp” function was conducted to eliminate batch effects and facilitate visualization, further assessing and validating key genes distinguishing IPF from healthy control samples.

### 2.3 eQTL analysis of exposure data

For identifying genetic variants linked to gene expression, eQTL analysis was conducted, leveraging transcriptome and genotype data from various cohorts. The most extensive meta-analysis of eQTL data to date, conducted by Westra et al., incorporated peripheral blood eQTL data from 5,311 European individuals (11). Summary eQTL data utilized in this study were retrieved from the GWAS Catalog website (https://gwas.mrcieu.ac.uk/). The R package “TwoSampleMR” was employed to identify strongly associated SNPs (p<5e-08) as instrumental variables. Linkage disequilibrium parameters were set at r2 < 0.001 and clumping distance = 10,000 kb. SNPs with weak associations or insufficient explanation of phenotypic variance were excluded, applying a filter of “F-test value >10”.

### 2.4 Determination of outcome data

Outcome data were derived from the genetic association database of the GWAS summary dataset (IEU) (https://gwas.mrcieu.ac.uk/). The GWAS ID used was finn-b-IPF, involving 1,028 cases and 196,986 European ancestry controls, including 16,380,413 SNPs. All GWAS summary statistics used in this study are publicly available and free to download. Ethical approval was obtained through the original analysis.

Outcome data were sourced from the genetic association database of the GWAS summary dataset (IEU) available at GWAS Catalog (https://gwas.mrcieu.ac.uk/). The specific GWAS ID used was finn-b-IPF, which included 1,028 cases and 196,986 controls of European ancestry, encompassing 16,380,413 SNPs. All GWAS summary statistics referenced in this study are publicly accessible and downloadable. Ethical clearance for this study was granted based on the original analyses conducted.

### 2.5 MR analysis

MR analysis was performed using the “TwoSampleMR” software package. The inverse variance-weighted (IVW) method was employed to investigate the relationship between specific genes and idiopathic pulmonary fibrosis. Additional sensitivity analyses were carried out using MR-Egger, simple mode, weighted median, and weighted mode methodologies (12, 13). Disease-related genes were identified using a threefold criteria approach:1. Genes demonstrating a P-value of less than 0.05 in the IVW method were initially selected.2. Genes were further refined based on the consistency of the direction of MR analysis results (Odds Ratio values) across three different methods.3. Genes exhibiting signs of pleiotropy with a P-value of less than 0.05 were excluded from the selection.

Following this process, co-expressed genes among these disease-related genes and the DEGs, encompassing both up-regulated and down-regulated genes, were identified through intersection. All intersecting genes were then individually subjected to MR analyses analysis to ascertain their causal links to the disease. This analysis included heterogeneity tests, pleiotropy tests, and leave-one-out sensitivity analyses, to evaluate the robustness and reliability of the results. To visually represent and support these findings, scatter plots, forest plots, and funnel plots were created and analyzed.

### 2.6 GO/KEGG enrichment analysis

The “clusterProfiler” R package was used for GO functional annotation and KEGG pathway enrichment analysis of co-expressed genes to understand potential functional pathways and pathogenesis mechanisms. The “clusterProfiler” package is an ontology-based tool for biological term classification and gene cluster enrichment analysis, with the study’s filtering criterion set at Pval<0.05.

### 2.7 Immune cell analysis

CIBERSORT analysis was employed to assess the infiltration levels of 22 types of immune cells in idiopathic pulmonary fibrosis (14), exploring the correlation between co-expressed genes in IPF and immune cell infiltration, and further investigating the regulatory mechanisms of IPF co-expressed genes on immune cells.

### 2.8 GSEA enrichment analysis

GSEA was used to determine whether functions or pathways related to co-expressed genes were enriched at the top or bottom of the ranking, indicating upregulation or downregulation trends, respectively. GSEA enrichment analysis was further employed to explore the activity level of related functions or pathways in the gene expression group. In GSEA, a p-value < 0.05 was considered statistically significant.

### 2.9 Validation group differential analysis

R software (version 4.3.2) was used to read dataset GSE135065 (preprocessed using the same methods as before) to validate whether co-expressed genes exhibited differences between control and experimental groups, and to compare these findings with the results of our MR analyses analysis.

## 3 Results

### 3.1 Overview of the three GEO datasets

This study obtained three IPF microarray datasets from the GEO database as the experimental group. These three datasets together comprise 61 IPF patients and 18 healthy controls. **Table 1** provides detailed information about the datasets included.

**Table 1:** Characteristics of the Three GEO Datasets.

GSE ID	Samples	Tissues	Platform	Experiment type	Last update date
GSE24206	17 cases and 6 controls	lung tissue	GPL570	Array	Mar 25, 2019
GSE53845	40 cases and 8 controls	lung tissue	GPL6480	Array	Jan 23, 2019
GSE195770	4 cases and 4 controls	lung tissue	GPL20844	Array	Jan 03, 2023

**Figure 1:**
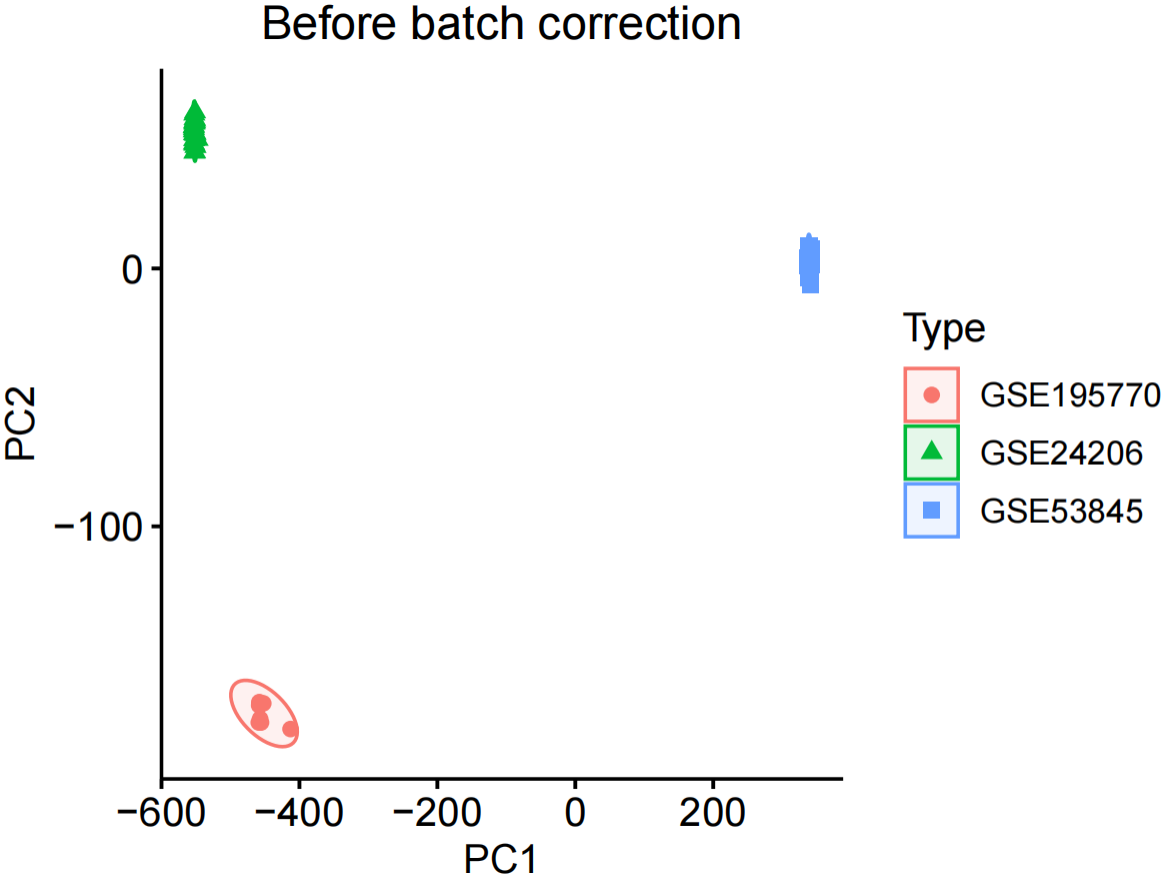
Before batch correction.

**Figure 2:**
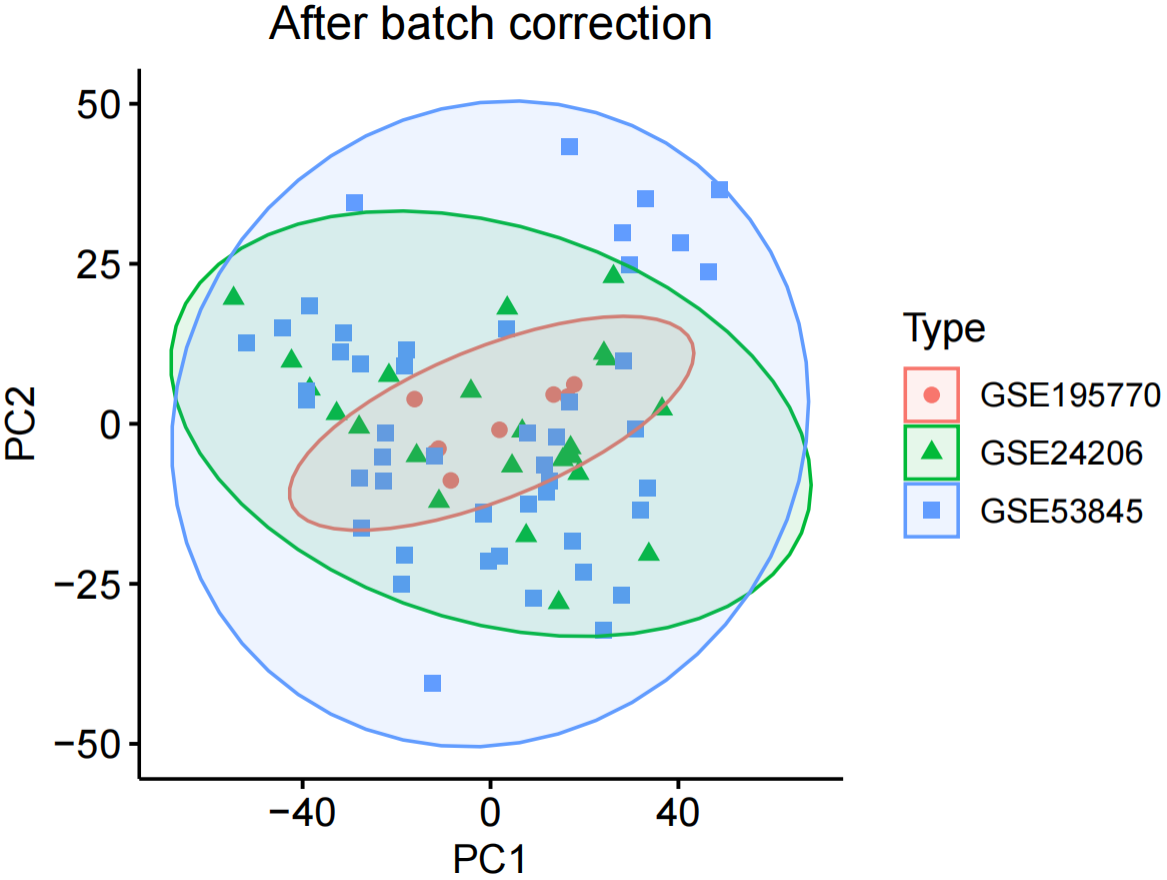
After batch correction.

We corrected and merged the expression values of each gene in its respective dataset using R version 4.3.2, and eliminated batch effects through Principal Component Analysis (PCA). As shown in **Figure 1**, the batch effects in the three IPF gene datasets are evident. After correction, as displayed in **Figure 2**, all samples in the dataset achieved acceptable homogeneity following PCA analysis.

### 3.2 DEGs identification

**Figure 3:**
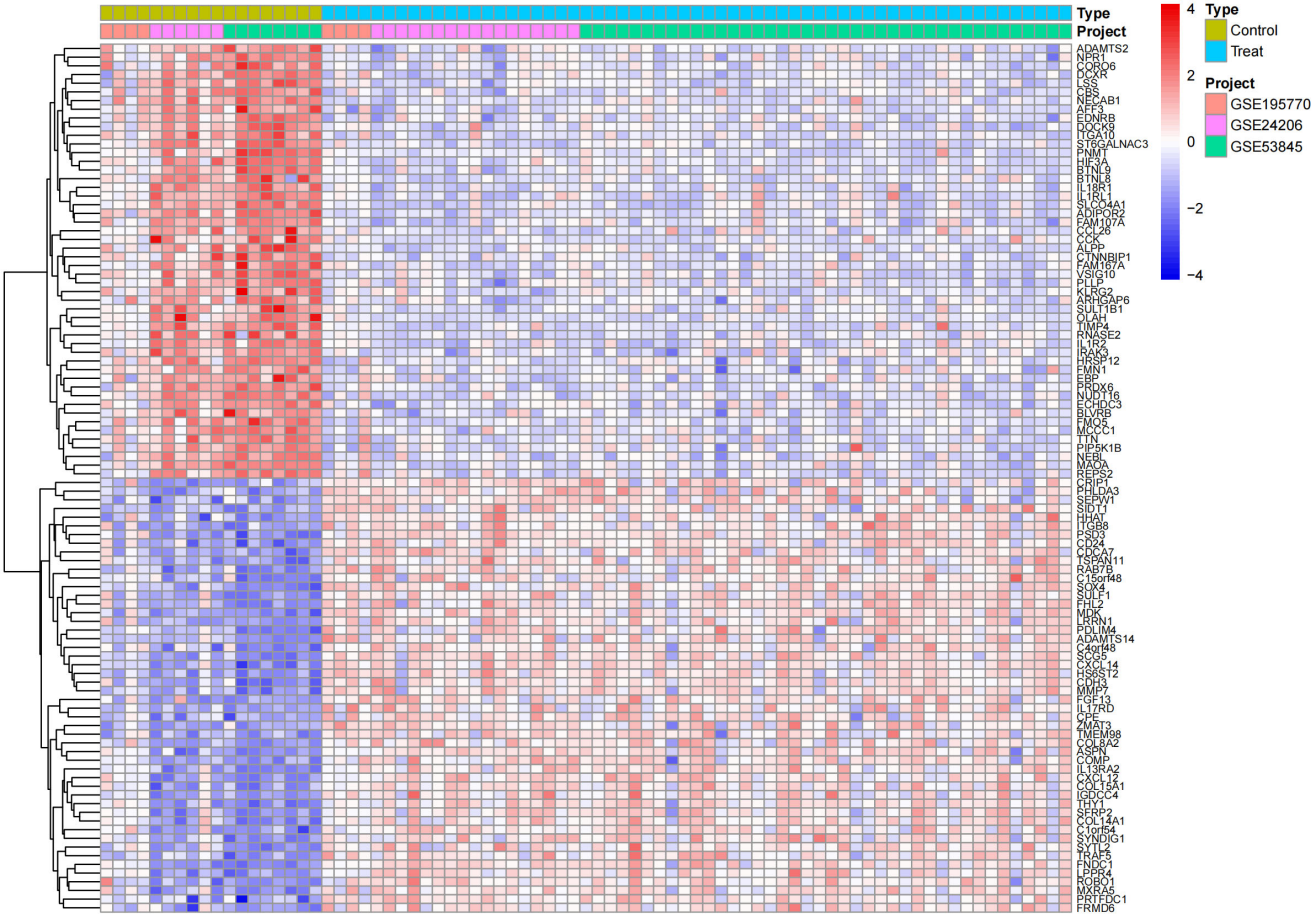
Differential gene expression heatmap.

**Figure 4:**
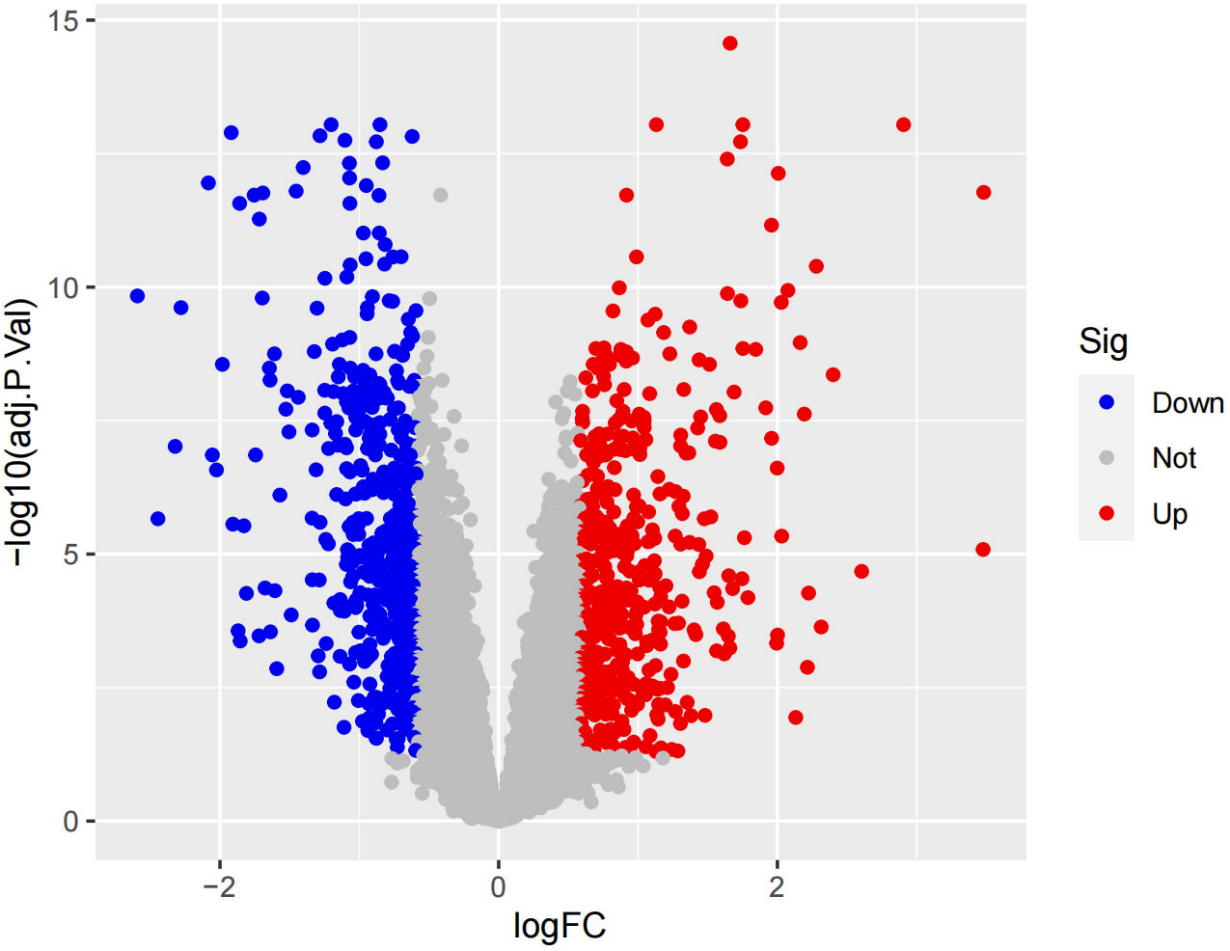
Volcano plo.

In the obtained results, the smaller the p-value, the higher the reliability of gene ranking and differential gene expression. Ultimately, we detected 486 up-regulated DEGs and 468 down-regulated DEGs. **Supplementary Table S1** provides detailed information on these significantly differentially expressed genes. The heatmap of DEGs expression in **Figure 3** displays the top 50 up-regulated DEGs and the top 50 down-regulated DEGs. The volcano plot of the integrated GEO dataset is shown in **Figure 4**.

### 3.3 MR analysis

After screening, we ultimately obtained 26,152 SNPs as instrumental variables, all of which adhered to the three basic assumptions of MR, and all selected SNPs had F-statistics exceeding 10 (**Supplementary Table S2** provides detailed information on the included data).

**Figure 5:**
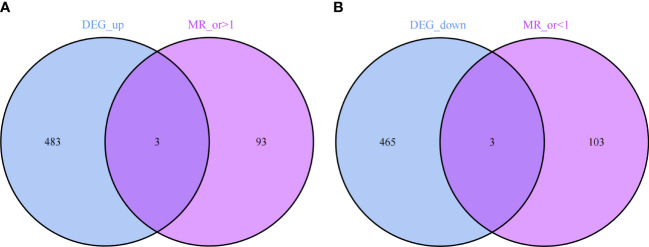
**(A)** 3 up-regulated co-expressed genes. **(B)** 3 down-regulated co-expressed genes.

Through the results of MR analysis and the three established filtering criteria, we identified 202 IPF-related genes (**Supplementary Table S3** provides detailed information on the data included). Further by intersecting, we obtained co-expressed genes between disease-related genes and DEGs, including 3 up-regulated genes (C12orf75, SPP1, ZG16B) and 3 down-regulated genes (LIN7A, PPP1R14A, TLR2), as shown in **Figures 5A, B**.

Subsequently, we conducted MR analyses on these six co-expressed genes with IPF to determine the causal effect of each gene on the disease.

The results revealed that in the MR analysis using the inverse-variance weighted method, all three up-regulated co-expressed genes showed a significant positive causal relationship with IPF. Specifically, C12orf75 (OR=1.162; 95% CI:[1.000 to 1.349]; P = 0.049), SPP1 (OR=1.221; 95% CI:[1.072 to 1.392]; P = 0.003), and ZG16B (OR=1.215; 95% CI:[1.018 to 1.451]; P = 0.031) demonstrated this relationship. Conversely, all three down-regulated co-expressed genes showed a significant negative causal relationship with IPF, namely LIN7A (OR=0.836; 95% CI:[0.701 to 0.997]; P = 0.046), PPP1R14A (OR=0.812; 95% CI:[0.673 to 0.980]; P = 0.030), and TLR2 (OR=0.580; 95% CI:[0.380 to 0.886]; P = 0.012).

**Figure 6:**
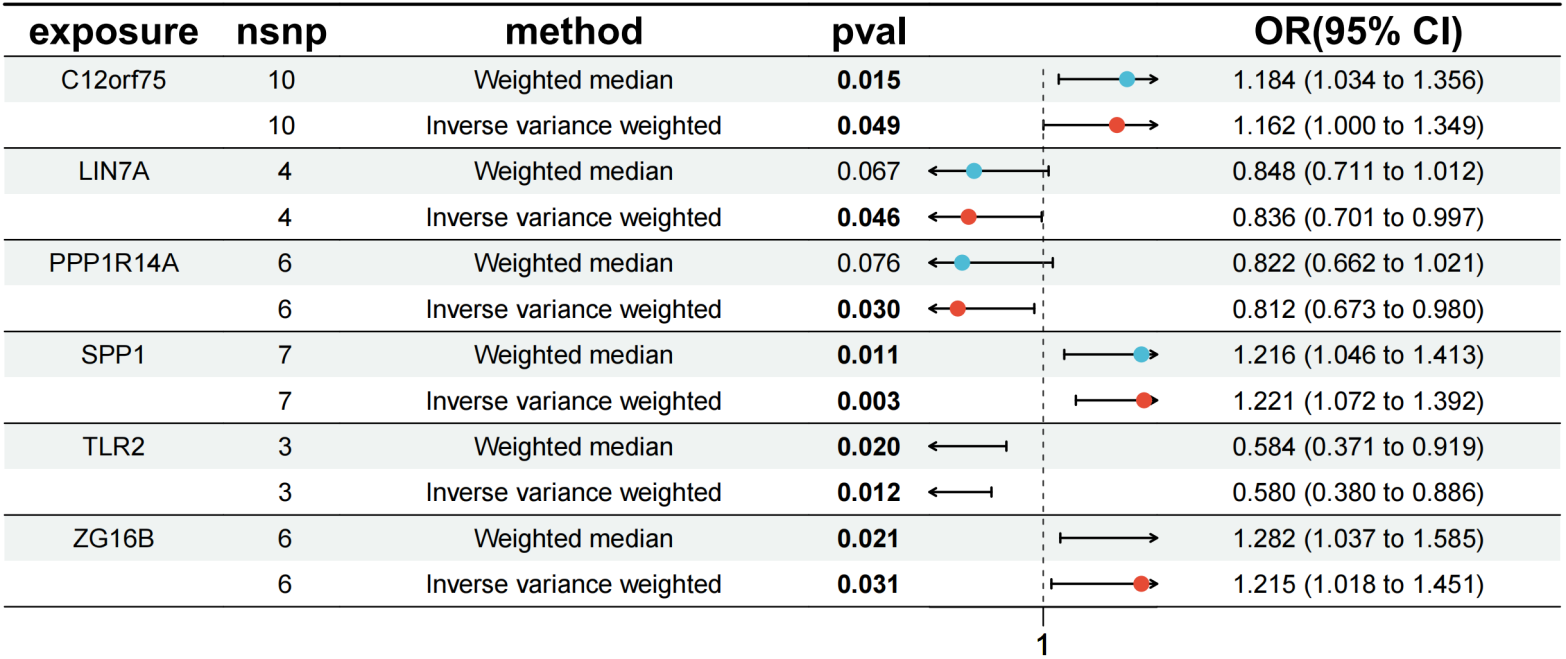
MR forest plot of co-expressed genes.

**Figure 7:**
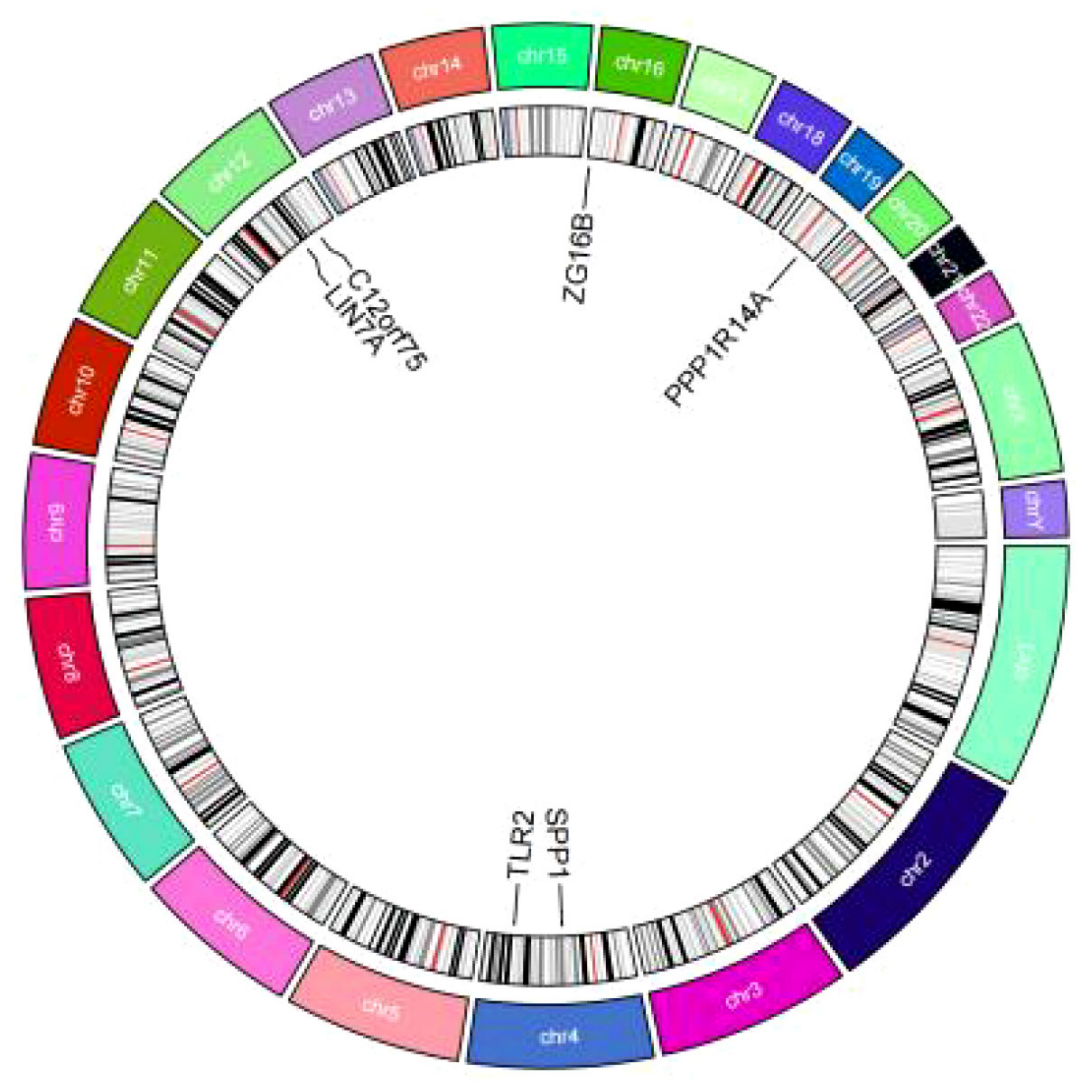
Circos plot of co-expressed genes.

In addition to MR-Egger, simple mode, weighted median, and weighted mode were used for further validation. Apart from the simple mode in C12orf75, simple mode and MR Egger in SPP1 and ZG16B, simple mode, MR Egger, and weighted mode in TLR2, and simple mode, MR Egger, weighted mode, and weighted median in PPP1R14A and LIN7A, the other methods had significant effects on these six genes. All methods for the three up-regulated genes consistently indicated an increase in IPF risk (OR > 1), while all methods for the three down-regulated genes consistently indicated a decrease in IPF risk (OR < 1) (**Figure 6**). It was found that the results of the heterogeneity tests and pleiotropy tests for the co-expressed genes all suggested P > 0.05, indicating no statistical significance and no need to consider the impact of heterogeneity and pleiotropy on the results. The leave-one-out sensitivity analysis showed that the effect sizes of the included IVs were close to the overall effect size, demonstrating the robustness of the analysis. Detailed information for each gene, including scatter plots, forest plots, funnel plots, and leave-one-out sensitivity analyses, can be found in **Supplementary Figure S1**. To further clarify the chromosomal distribution of the aforementioned genes, we visualized the co-expressed genes (**Figure 7**).

### 3.4 GO and KEGG enrichment analysis

**Figure 8:**
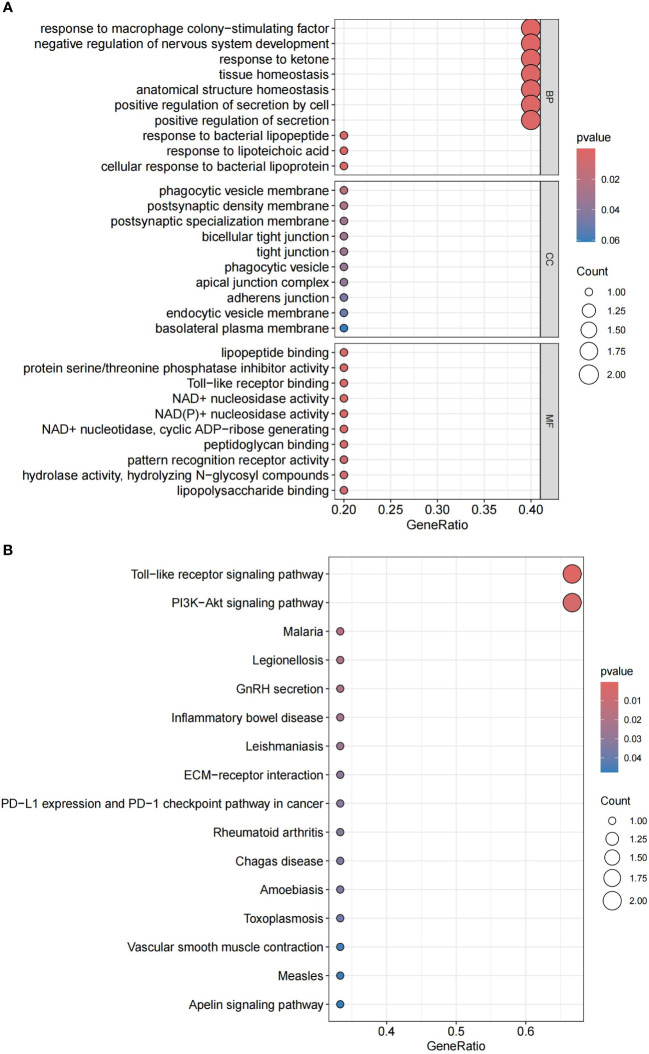
**(A)** GO enrichment analysis of candidate hub genes. **(B)** KEGG enrichment analysis of candidate hub genes.

Through GO and KEGG analysis, we further explored the potential roles of these six co-expressed genes (**Figures 8A, B**). GO enrichment analysis indicated that the co-expressed genes mainly affect biological functions such as response to macrophage colony-stimulating factor, negative regulation of nervous system development, response to ketone, tissue homeostasis, anatomical structure homeostasis, and positive regulation of secretion. KEGG enrichment analysis revealed that the co-expressed genes primarily impact the Toll-like receptor signaling pathway and the PI3K-Akt signaling pathway. Detailed data can be found in **Supplementary Table S4**.

### 3.5 Assessment of immune cell infiltration in idiopathic pulmonary fibrosis

**Figure 9:**
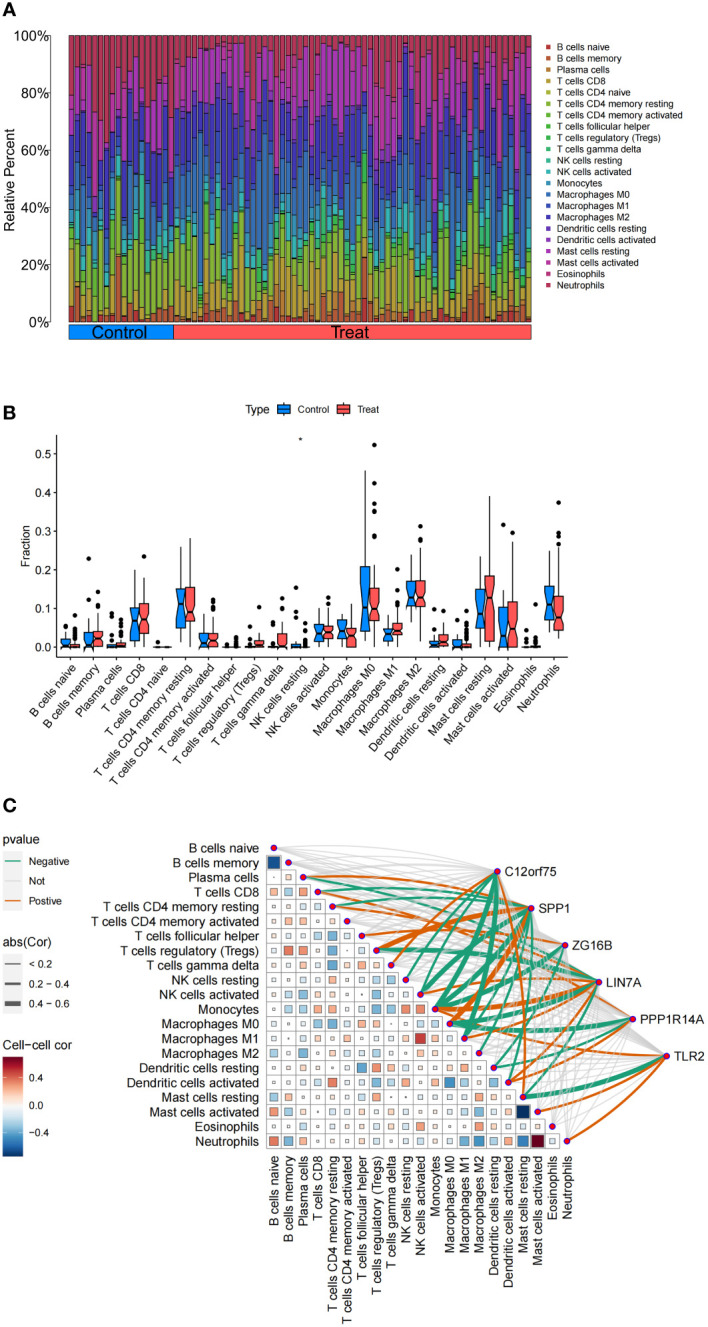
Analysis of Immune Cell Infiltration in IPF. **(A)** Stacked histogram of the proportions of immune cells between the IPF group and the control group. **(B)** Box plot showing the comparison of 22 types of immune cells between the IPF group and the control group. **(C)** Heatmap showing the correlation between 22 types of immune cells and co-expressed genes. *p<0.05.

The functional and pathway analysis of co-expressed genes in IPF shows a close relationship with inflammatory and immune processes. The CIBERSORT algorithm was used to infer immune cell characteristics and to explore the correlation between co-expressed genes in IPF and immune cell infiltration. **Figure 9A** displays the proportions of 22 types of immune cells in each sample. We observed a significant difference in a specific immune cell subtype (NK cells resting) between the IPF and control group samples. Specifically, the proportion of NK cells resting was significantly lower in IPF patient samples compared to the control group (**Figure 9B**).

Additionally, the correlation analysis with 22 types of immune cells indicates (**Figure 9C**) that the co-expressed gene C12orf75 is positively correlated with T cells gamma delta and Mast cells resting, and negatively correlated with NK cells resting, NK cells activated, Monocytes, and Macrophages M1. The co-expressed gene SPP1 is positively correlated with Plasma cells, Tregs, and Macrophages M0, and negatively correlated with T cells CD8, T cells CD4 memory resting, NK cells activated, Monocytes, Macrophages M1, Macrophages M2, and Dendritic cells activated. The co-expressed gene ZG16B is positively correlated with T cells follicular helper and negatively correlated with Monocytes and Dendritic cells resting. The co-expressed gene LIN7A is positively correlated with T cells CD4 memory resting, NK cells activated, Monocytes, Macrophages M1, and Dendritic cells activated, and negatively correlated with Plasma cells, Tregs, Macrophages M0, and Mast cells resting. The co-expressed gene PPP1R14A is positively correlated with T cells CD8 and Dendritic cells activated, and negatively correlated with Macrophages M0 and Dendritic cells resting. The co-expressed gene TLR2 is positively correlated with Monocytes, Mast cells activated, and Neutrophils, and negatively correlated with Mast cells resting.

### 3.6 GSEA enrichment analysis

**Figure 10:**
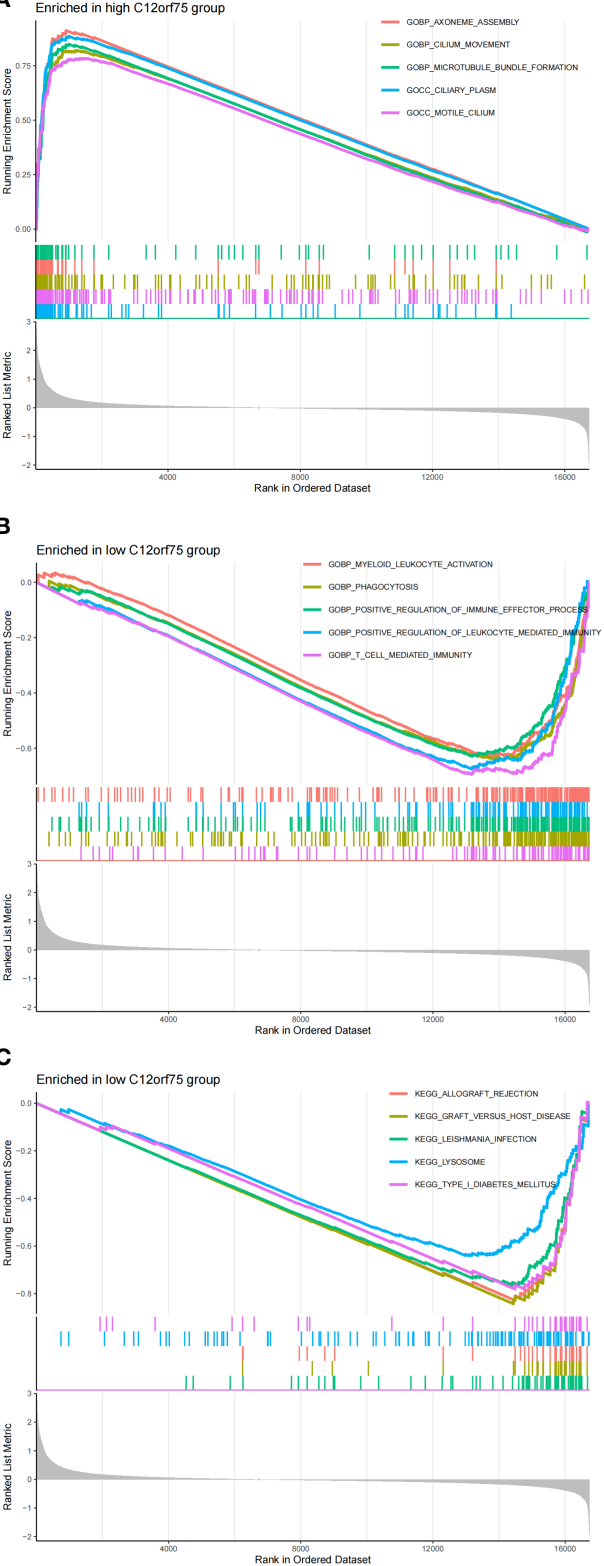
Differential impact of C12orf75 expression on biological functions and pathways in IPF. **(A)** The top 5 active biological functions in the C12orf75 high expression group. **(B)** The top 5 active biological functions in the C12orf75 low expression group. **(C)** The top 5 active pathways in the low C12orf75 expression group.

We found that the co-expressed up-regulated gene C12orf75 has a negative regulatory relationship with the immune cells NK cells resting, and compared to the control group, the proportion of NK cells resting in IPF is lower. Therefore, we further explored the activity level of related functions or pathways in this gene expression group using GSEA enrichment analysis. The results revealed that the top 5 active biological functions in the C12orf75 high expression group are Axoneme Assembly, Cilium Movement, Microtubule Bundle Formation, Ciliary Plasm, and motile cilium (**Figure 10A**). The top five active biological functions in the C12orf75 low expression group are Myeloid Leukocyte Activation, Phagocytosis, Positive regulation of immune effector process, Positive regulation of Leukocyte Mediated Immunity, and T cell mediated immunity (**Figure 10B**). The top five active pathways in the C12orf75 low expression group are Allograft Rejection, Graft Versus Host Disease, Leishmania infection, Lysosome, and Type I Diabetes Mellitus (**Figure 10C**). No active pathways were found in the C12orf75 high expression group.

### 3.7 Validation group differential analysis

**Figure 11:**
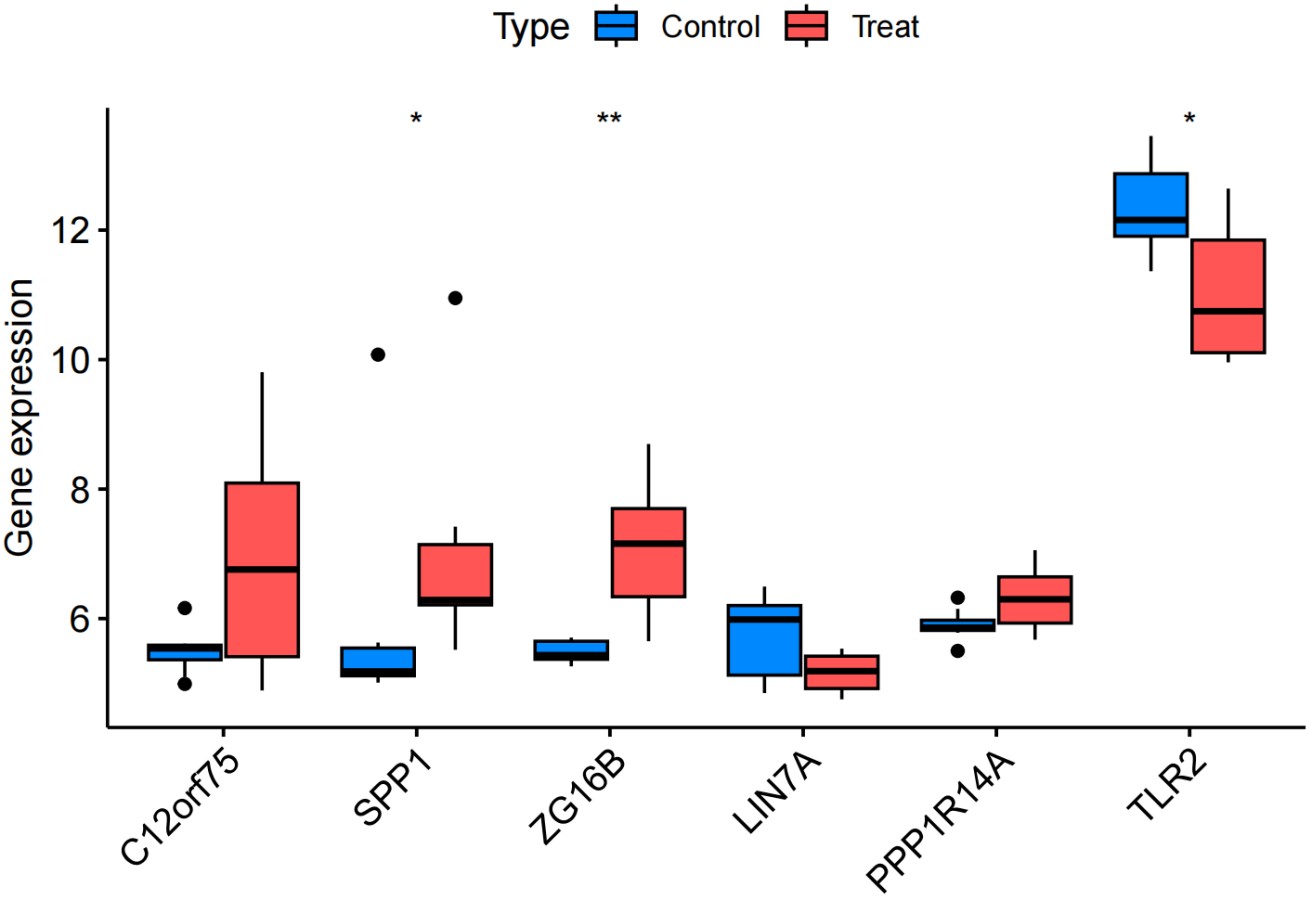
Validation group differential analysis. * p<0.05; ** p<0.01.

We confirmed the co-expressed genes found in the MR analysis to have the appropriate levels of expression. The findings revealed that IPF samples had higher expressions of C12orf75, SPP1, and ZG16B than the healthy control group did. SPP1 and ZG16B had significantly higher expressions (P<0.05 and P<0.01, respectively). Meanwhile, the expressions of LIN7A and TLR2 were down-regulated in IPF samples compared to the healthy control group, with significant expression of TLR2 (P<0.05) (**Figure 11**). Clearly, the expression levels of the three up-regulated and two down-regulated genes are consistent with the results proposed in our MR analysis, lending greater credibility to the MR results.

## 4 Discussion

IPF is a chronic, progressive pulmonary disease with an adverse prognosis, rendering it an incurable condition that severely impairs patient quality of life, posing a significant challenge in the current clinical landscape. This study aimed to provide new insights into the molecular mechanisms of IPF through a comprehensive analysis of datasets from the GEO database, applying MR analysis, and assessing immune cell infiltration, thereby identifying potential targets for future therapeutic strategy development. We extracted three IPF microarray datasets from the GEO database, including 61 IPF patients and 18 healthy controls. Through an in-depth analysis of these datasets, we successfully identified specific DEGs that might be crucial to the pathophysiology of IPF. Our detailed analysis delineated a range of specific DEGs that could be central to the pathogenesis of IPF. This methodology aligns with the research conducted by Dai et al. (15), but extends their work by providing further in-depth MR causal validation for the identified DEGs.

This study intersected 486 up-regulated and 468 down-regulated DEGs with IPF related genes identified through MR analysis, ultimately identifying six co-expressed target genes significantly associated with IPF. This includes three up-regulated genes (C12orf75, SPP1, ZG16B) and three down-regulated genes (LIN7A, PPP1R14A, TLR2). The expression patterns of these genes provide new insights into the genetic basis of IPF, potentially reflecting key molecular changes in the disease pathology. Further MR analysis confirmed that increased expressions of C12orf75, SPP1, and ZG16B elevate the risk of IPF, while reduced expressions of LIN7A, PPP1R14A, and TLR2 are also associated with an increased disease risk. Our research focuses on these newly identified gene targets, which may play a crucial role in the pathogenesis of IPF.

### Up-regulated gene

C12orf75: Located on chromosome 12, C12orf75 was found to be significantly up-regulated in IPF patients in our study, suggesting its potential role in the progression of IPF. While current research on the role of C12orf75 in IPF is limited, its expression pattern suggests a possible association with lung tissue repair and fibrotic processes. This aligns with the work of Richeldi et al., who emphasized the importance of targeting specific molecular pathways in IPF treatment (16). The limited current research on C12orf75 in IPF suggests that our findings might unveil a new avenue for investigation.

SPP1 (Osteopontin): Situated on chromosome 4, SPP1 is recognized as an inflammatory and fibrosis regulatory factor.It is extensively expressed in a wide range of cell types and is important for extracellular matrix (ECM) and intercellular communication (17). SPP1’s involvement in the pathological processes of IPF is well-documented (18). It is believed to participate in multiple biological functions, including cell adhesion, migration, survival, and immune regulation. In the context of IPF, SPP1’s upregulation may exacerbate pulmonary inflammation and fibrosis. Our research corroborates with findings from Zhang et al., indicating a link between SPP1 and heightened immune infiltration in IPF (19). The differential expression patterns observed in IPF, such as the increased expression of SPP1, appear to be critically involved in the disease’s pathogenesis. These observations are supported by experimental studies conducted by Morse et al., which identified three distinct macrophage subgroups in the IPF lung, one characterized by high SPP1 expression and pronounced proliferation (20). Macrophages with elevated SPP1 levels may thus play a pivotal role in IPF’s pulmonary fibrosis, particularly in the activation of myofibroblasts. SPP1 may be a useful biomarker for IPF patients’ diagnosis and prognosis, according to a meta-analysis of 13 studies (21). Given these insights, the specific function of SPP1 in the pathology of IPF warrants further exploration.

ZG16B (Zymogen Granule Protein 16B): Located on chromosome 16, the specific function of the ZG16B gene is not yet fully understood; it may play a role in exocrine tissues. Recent studies have found an upregulation of ZG16B in various cancers (22–24) and it plays a significant role in the development of diseases such as atherosclerosis (25). Mody et al. discovered that ZG16b protein is located in serous and seromucous acinar cells in single-cell RNA sequencing data of healthy human labial minor salivary glands (26), possibly related to mucosal secretion pathways, cell migration, and homeostatic markers. It may serve as a new biomarker for salivary gland dysfunction (27). However, current research on the direct association of ZG16B with IPF is limited, and our findings may reveal a new research direction.

### Down-regulated gene

LIN7A: Located on chromosome 12, LIN7A plays a crucial role in the nervous system and cell polarity. Matsumoto and others have found that LIN7A is key in brain development, and its deficiency may lead to intellectual disabilities and incomplete corpus callosum development (28). Its down-regulation in IPF might similarly impact cell signaling and tissue remodeling. Currently, there’s limited research on LIN7A’s role in IPF, offering a new perspective in understanding the cellular biology of IPF.

TLR2 (Toll-like Receptor 2): Situated on chromosome 4, TLR2 is essential for initiating innate immune responses because it can identify various molecular patterns linked to microbial pathogens. TLR2’s activation is crucial in combating infections through the stimulation of immune cells and promotion of inflammatory responses (29). Research has shown that respiratory epithelial cells exposed to TNF-α or corticosteroids exhibit a notable increase in TLR2 expression (30, 31), suggesting that TLR2 expression may be modulated by changes in the pulmonary cytokine environment due to tissue injury. In cardiovascular diseases, the downregulation of TLR2 expression in myocardial infarction models has demonstrated a protective effect (32). As an integral part of the immune system, TLR2’s involvement in IPF could be significant, particularly in terms of inflammation and immune regulation. The activation of TLR2 may facilitate the recruitment and activation of inflammatory cells, influencing IPF development (33). A study by Samara et al. suggests a critical role for TLR2 in IPF’s immune regulation process (34), thereby highlighting the need for further research into its specific mechanisms in IPF.

Beyond the gene-specific effects, our research extends to the immunological dimension of IPF. To delve deeper into the modes of action of genes, we further explored the potential roles of these co-expressed genes, especially their importance in macrophage responses, regulation of nervous system development, and maintenance of tissue homeostasis, through GO and KEGG enrichment analysis. These analyses reveal key biological processes and pathways related to IPF, closely associated with inflammation and immune processes.

Using the CIBERSORT algorithm, we evaluated the distribution of various immune cell subgroups in IPF, thus illuminating the contribution of immune cells to the disease’s pathogenesis (35). This approach is in line with the growing scholarly interest in analyzing immune cells associated with IPF (36). Building upon these findings, we further investigated the connections between target genes of IPF and immune cell infiltration, and the specific regulatory effects these genes exert on immune cells. Our results align with existing research on IPF, reinforcing the importance of immune cells in the disease’s development (33). They also offer insights into potential future therapeutic avenues, such as modulating specific immune cell activities to decelerate or reverse the progression of IPF (37). This aspect of the research not only corroborates existing knowledge but also opens new doors for targeted treatment strategies in IPF.

PPP1R14A (Protein Phosphatase 1 Regulatory Inhibitor Subunit 14A): Located on chromosome 19, PPP1R14A belongs to the Protein Phosphatase 1 (PP1) inhibitor family and is responsible for encoding CPI-17 (C-kinase-activated PP1 inhibitor, 17kDa). CPI-17 regulates various cellular functions by inhibiting PP1 activity, especially in smooth muscle cell contraction. Past research indicates that PPP1R14A plays a crucial role in the development and progression of some tumors (38–40). Current research on PPP1R14A’s role in IPF is limited, necessitating further investigation.

We found that in the co-expressed up-regulated genes, C12orf75 has a negative regulatory relationship with the immune cells NK cells resting, and compared to the control group, the proportion of NK cells resting in IPF is significantly reduced. Therefore, through further GSEA enrichment analysis, we discovered that C12orf75 appears to play multifaceted roles in the pathogenesis of IPF. Its expression levels may be related to changes in immune cell activity and biological functions associated with ciliary processes and autoimmune responses. C12orf75 may play a role in regulating the immune response in IPF, particularly by affecting NK cell activity and leading to an imbalance in the immune environment. The high expression of C12orf75 might be associated with ciliary dysfunction, potentially disrupting normal lung epithelial cell function and leading to the fibrotic process. The active pathways in the low expression group of C12orf75 indicate the presence of autoimmune components in IPF, potentially mediated by the regulatory action of C12orf75.

The consistency of the MR analysis with the validation group’s differential analysis results confirms the robustness of our MR findings, enhancing the credibility of these genes’ association with IPF disease. The upregulation of SPP1 and ZG16B may promote inflammatory responses in IPF, potentially being key driving factors in the development of IPF. The downregulation of TLR2 may lead to dysregulation of the immune response, thereby promoting the progression of IPF, while the downregulation of LIN7A may affect intercellular signal transmission, leading to abnormal lung tissue repair processes, and thus promoting fibrosis. Certainly, newly identified therapeutic targets in recent research also warrant further attention. For example, research teams have found through basic experiments that MKP-5 expression was increased in lung fibroblasts derived from IPF. Consequently, inhibiting MKP-5 could potentially serve as a novel therapeutic target for IPF treatment (41). Autotaxin (ATX) may represent another potential therapeutic target for idiopathic pulmonary fibrosis. Research teams, through a comprehensive literature review, have discovered that targeting ATX not only offers a novel approach to IPF treatment but also highlights its relevance in the pathogenesis of both IPF and COVID-19 related pulmonary complications. This suggests that ATX could be a potential common therapeutic target, warranting further investigation (42).

However, these hypotheses necessitate further experimental validation to clarify how these genes influence these pathways and contribute to IPF progression. This includes validation in larger independent cohorts and more comprehensive functional studies before clinical applications are considered. It’s also important to recognize potential limitations such as selection bias and the inherent constraints of the statistical methods and bioinformatics tools used in our study.

## 5 Conclusions

This study provides a detailed analysis of Idiopathic Pulmonary Fibrosis, identifying key genes and pathways using advanced bioinformatics and statistical methods. It emphasizes the importance of immune cells and genetic factors, focusing on genes such as C12orf75, SPP1, ZG16B, LIN7A, PPP1R14A and TLR2. The findings provide insights into the complex molecular mechanisms of IPF and offer avenues for novel therapeutic interventions. However, these results require further validation and consideration of the inherent limitations in data selection and analysis techniques. Overall, the study contributes to a deeper understanding of IPF, indicating the need for targeted molecular and immunological approaches in future treatment and research strategies.

## Data availability statement

Summary statistics of genetic instruments can be obtained from the websites listed on the referenced GWAS or GWAS meta-analysis.

## Ethics statement

All the GWAS/GWAS meta-analysis from which the summary statistics were extracted from had obtained ethics approval from the respective institutional review board. The studies were conducted in accordance with the local legislation and institutional requirements. Written informed consent for participation was not required from the participants or the participants’ legal guardians/next of kin in accordance with the national legislation and institutional requirements.

## Author contributions

WH: Data curation, Software, Visualization, Writing – original draft, Writing – review & editing. YX: Conceptualization, Data curation, Methodology, Supervision, Visualization, Writing – original draft.

## Supplementary material

Supplementary Figure 1 Scatterplots, forest plots, funnel plots and leave-one-out sensitivity analysis plots for six target genes.

Supplementary Table SN Results of MR analysis, heterogeneity and pleiotropy tests for six co-expressed genes.

Supplementary Table S1 DEGs identification.

Supplementary Table S2 Instrument variables for exposure.

Supplementary Table S3 IPF related genes screened by MR.

Supplementary Table S4 GO and KEGG enrichment results.

## References

[1] Blackwell TS Tager AM Borok Z Moore BB Schwartz DA Anstrom KJ. Future directions in idiopathic pulmonary fibrosis research. An NHLBI workshop report. Am. J Respir Crit Care Med. (2014) 189:214–22. doi: 10.1164/rccm.201306-1141WS PMC3983890 24160862

[2] Yanagihara T Sato S Upagupta C Kolb M. What have we learned from basic science studies on idiopathic pulmonary fibrosis? Eur Respir Rev. (2019) 28:190029. doi: 10.1183/16000617.0029-2019 31511255 PMC9488501

[3] Phan THG Paliogiannis P Nasrallah GK Giordo R Eid AH Fois AG. Emerging cellular and molecular determinants of idiopathic pulmonary fibrosis. Cell Mol Life Sci. (2021) 78:2031–57. doi: 10.1007/s00018-020-03693-7 PMC7669490 33201251

[4] Lehmann M Korfei M Mutze K Klee S Skronska-Wasek W Alsafadi HN. Senolytic drugs target alveolar epithelial cell function and attenuate experimental lung fibrosisex vivo. Eur Resp J. (2017) 50:1602367. doi: 10.1183/13993003.02367-2016 PMC5593348 28775044

[5] Ma H Liu S Li S Xia Y. Targeting growth factor and cytokine pathways to treat idiopathic pulmonary fibrosis. Front Pharmacol. (2022) 13:918771. doi: 10.3389/fphar.2022.918771 35721111 PMC9204157

[6] Yang DC Li JM Xu J Oldham J Phan SH Last JA. Tackling MARCKS-PIP3 circuit attenuates fibroblast activation and fibrosis progression. FASEB J. (2019) 33:14354–69. doi: 10.1096/fj.201901705R PMC6894092 31661644

[7] Heukels P Moor CC von der Thusen JH Wijsenbeek MS Kool M. Inflammation and immunity in IPF pathogenesis and treatment. Respir Med. (2019) 147:79–91. doi: 10.1016/j.rmed.2018.12.015 30704705

[8] King TJ Pardo A Selman M. Idiopathic pulmonary fibrosis. Lancet. (2011) 378:1949–61. doi: 10.1016/S0140-6736(11)60052-4 21719092

[9] Antoniou KM Wuyts W Wijsenbeek M Wells AU. Medical therapy in idiopathic pulmonary fibrosis. Semin. Respir. Crit Care Med. (2016) 37:368–77. doi: 10.1055/s-00000075 27231861

[10] Karampitsakos T Vraka A Bouros D Liossis SN Tzouvelekis A. Biologic treatments in interstitial lung diseases. Front Med. (2019) 6:41. doi: 10.3389/fmed.2019.00041 PMC6425869 30931306

[11] Westra H Peters MJ Esko T Yaghootkar H Schurmann C Kettunen J. Systematic identification of trans eQTLs as putative drivers of known disease associations. Nat Genet. (2013) 45:1238–43. doi: 10.1038/ng.2756 PMC3991562 24013639

[12] Dudbridge F. Polygenic mendelian randomization. Cold Spring Harb. Perspect Med. (2021) 11:a39586. doi: 10.1101/cshperspect.a039586 PMC7849343 32229610

[13] Emdin CA Khera AV Kathiresan S. Mendelian randomization. Jama-J.Am Med Assoc. (2017) 318:1925–6. doi: 10.1001/jama.2017.17219 29164242

[14] Chen B Khodadoust MS Liu CL Newman AM Alizadeh AA. Profiling tumor infiltrating immune cells with CIBERSORT. Methods Mol Biol. (2018) 1711:243–59. doi: 10.1007/978-1-4939-7493-1_12 PMC5895181 29344893

[15] Dai X Yang Z Zhang W Liu S Zhao Q Liu T. Identification of diagnostic gene biomarkers related to immune infiltration in patients with idiopathic pulmonary fibrosis based on bioinformatics strategies. Front Med. (2022) 9:959010. doi: 10.3389/fmed.2022.959010 PMC9729277 36507532

[16] Richeldi L du Bois RM Raghu G Azuma A Brown KK Costabel U. Efficacy and safety of nintedanib in idiopathic pulmonary fibrosis. N Engl J Med. (2014) 370:2071–82. doi: 10.1056/NEJMoa1402584 24836310

[17] Yim A Smith C Brown AM. Osteopontin/secreted phosphoprotein-1 harnesses glial-, immune-, and neuronal cell ligand-receptor interactions to sense and regulate acute and chronic neuroinflammation. Immunol Rev. (2022) 311:224–33. doi: 10.1111/imr.13081 PMC9790650 35451082

[18] Ley B Brown KK Collard HR. Molecular biomarkers in idiopathic pulmonary fibrosis. Am J Physiol-Lung Cell Mol Physiol. (2014) 307:L681–91. doi: 10.1152/ajplung.00014.2014 PMC4280147 25260757

[19] Zhang Y Wang C Xia Q Jiang W Zhang H Amiri-Ardekani E. Machine learning-based prediction of candidate gene biomarkers correlated with immune infiltration in patients with idiopathic pulmonary fibrosis. Front Med. (2023) 10:1001813. doi: 10.3389/fmed.2023.1001813 PMC9968813 36860337

[20] Morse C Tabib T Sembrat J Buschur KL Bittar HT Valenzi E. Proliferating SPP1/MERTK-expressing macrophages in idiopathic pulmonary fibrosis. Eur Resp J. (2019) 54:1802441. doi: 10.1183/13993003.02441-2018 PMC8025672 31221805

[21] Liao Y Wang R Wen F. Diagnostic and prognostic value of secreted phosphoprotein 1 for idiopathic pulmonary fibrosis: a systematic review and meta-analysis. Biomarkers. (2023) 28:87–96. doi: 10.1080/1354750X.2022.2148744 36377416

[22] Li L Zhang Y Ren Y Cheng Z Zhang Y Wang X. Pan-cancer single-cell analysis reveals the core factors and pathway in specific cancer stem cells of upper gastrointestinal cancer. Front Bioeng Biotechnol. (2022) 10:849798. doi: 10.3389/fbioe.2022.849798 35646860 PMC9136039

[23] Lu H Shi C Liu X Liang C Yang C Wan X. Identification of ZG16B as a prognostic biomarker in breast cancer. Open Med. (2021) 16:1–13. doi: 10.1515/med-2021-0004 PMC7718615 33336077

[24] Escudero-Paniagua B Bartolomé RA Rodríguez S De Los Ríos V Pintado L Jaén M. PAUF/ZG16B promotes colorectal cancer progression through alterations of the mitotic functions and the Wnt/β-catenin pathway. Carcinogenesis. (2019) 41:203–13. doi: 10.1093/carcin/bgz093 31095674

[25] Martin-Lorenzo M Zubiri I Maroto AS Gonzalez-Calero L Posada-Ayala M de la Cuesta F. KLK1 and ZG16B proteins and arginine–proline metabolism identified as novel targets to monitor atherosclerosis, acute coronary syndrome and recovery. Metabolomics. (2015) 11:1056–67. doi: 10.1007/s11306-014-0761-8 PMC4573654 26413039

[26] Mody DP Da Silva AC Sharma R Mays J. Characterization of ZG16b protein exocrine function and biomarker potential for salivary gland damage in chronic graft vs. host disease. J Immunol. (2023) 210:118–73. doi: 10.4049/jimmunol.210.Supp.173.18

[27] Costa-da-Silva AC Aure MH Dodge J Martin D Dhamala S Cho M. Salivary ZG16B expression loss follows exocrine gland dysfunction related to oral chronic graft-versus-host disease. Iscience. (2022) 25:103592. doi: 10.1016/j.isci.2021.103592 35005541 PMC8718990

[28] Matsumoto A Mizuno M Hamada N Nozaki Y Jimbo EF Momoi MY. LIN7A depletion disrupts cerebral cortex development, contributing to intellectual disability in 12q21-deletion syndrome. PloS One. (2014) 9:e92695. doi: 10.1371/journal.pone.0092695 24658322 PMC3962435

[29] Beg AA. Endogenous ligands of Toll-like receptors: implications for regulating inflammatory and immune responses. Trends Immunol. (2002) 23:509–12. doi: 10.1016/S1471-4906(02)02317-7 12401394

[30] Armstrong L Medford ARL Uppington KM Robertson J Witherden IR Tetley TD. Expression of functional toll-like receptor-2 and -4 on alveolar epithelial cells. Am J Respir Cell Mol Biol. (2004) 31:241–5. doi: 10.1165/rcmb.2004-0078OC 15044215

[31] Homma T Kato A Hashimoto N Batchelor J Yoshikawa M Imai S. Corticosteroid and cytokines synergistically enhance toll-like receptor 2 expression in respiratory epithelial cells. Am J Respir Cell Mol Biol. (2004) 31:463–9. doi: 10.1165/rcmb.2004-0161OC 15242847

[32] Wang S Xuan L Hu X Sun F Li S Li X. LncRNA CCRR attenuates post-myocardial infarction inflammatory response by inhibiting the TLR signaling pathway. Can J Cardiol. (2023). doi: 10.1016/j.cjca.2023.12.003 38081511

[33] George PM Spagnolo P Kreuter M Altinisik G Bonifazi M Martinez FJ. Progressive fibrosing interstitial lung disease: clinical uncertainties, consensus recommendations, and research priorities. Lancet Resp Med. (2020) 8:925–34. doi: 10.1016/S2213-2600(20)30355-6 32890499

[34] Samara K. Expression profiles of Toll-like receptors in non-small cell lung cancer and idiopathic pulmonary fibrosis. Int J Oncol. (2012) 40:1397–404. doi: 10.3892/ijo 22344343

[35] Vancheri C Failla M Crimi N Raghu G. Idiopathic pulmonary fibrosis: a disease with similarities and links to cancer biology. Eur Resp J. (2010) 35:496–504. doi: 10.1183/09031936.00077309 20190329

[36] Wu Z Chen H Ke S Mo L Qiu M Zhu G. Identifying potential biomarkers of idiopathic pulmonary fibrosis through machine learning analysis. Sci Rep. (2023) 13:16559. doi: 10.1038/s41598-023-43834-z 37783761 PMC10545744

[37] Flaherty KR Wells AU Cottin V Devaraj A Walsh S Inoue Y. Nintedanib in progressive fibrosing interstitial lung diseases. N Engl J Med. (2019) 381:1718–27. doi: 10.1056/NEJMoa1908681 31566307

[38] Xu J Zhang Y Shi Y Yin D Dai P Zhao W. CPI-17 overexpression and its correlation with the NF2 mutation spectrum in sporadic vestibular schwannomas. Otol Neurotol. (2020) 41:e94–e102. doi: 10.1097/MAO.0000000000002430 31789805

[39] Riecken LB Zoch A Wiehl U Reichert S Scholl I Cui Y. CPI-17 drives oncogenic Ras signaling in human melanomas via Ezrin-Radixin-Moesin family proteins. Oncotarget. (2016) 7:78242–54. doi: 10.18632/oncotarget.v7i48 PMC5346635 27793041

[40] Hagel C Dornblut C Schulz A Wiehl U Friedrich RE Huckhagel T. The putative oncogene CPI-17 is up-regulated in schwannoma. Neuropathol. Appl Neurobiol. (2016) 42:664–8. doi: 10.1111/nan.12330 27248983

[41] Xylourgidis N Min K Ahangari F Yu G Herazo-Maya JD Karampitsakos T. Role of dual-specificity protein phosphatase DUSP10/MKP-5 in pulmonary fibrosis. Am J Physiol.-Lung Cell Mol Physiol. (2019) 317:L678–89. doi: 10.1152/ajplung.00264.2018 PMC6879900 31483681

[42] Ntatsoulis K Karampitsakos T Tsitoura E Stylianaki EA Matralis AN Tzouvelekis A. Commonalities between ARDS, pulmonary fibrosis and COVID-19: the potential of autotaxin as a therapeutic target. Front Immunol. (2021) 12:687397. doi: 10.3389/fimmu.2021.687397 34671341 PMC8522582

