# Supplementary material for: Transcriptomics in idiopathic pulmonary fibrosis unveiled: a new perspective from differentially expressed genes to therapeutic targets
Source: Front Immunol. 2024 Mar 19;15:1375171. doi: 10.3389/fimmu.2024.1375171 (PMC10985171; doi:10.3389/fimmu.2024.1375171)
Supplement: Supplementary Figure 1 — Scatterplots, forest plots, funnel plots and leave-one-out sensitivity analysis plots for six target genes. [file DataSheet_1.pdf]

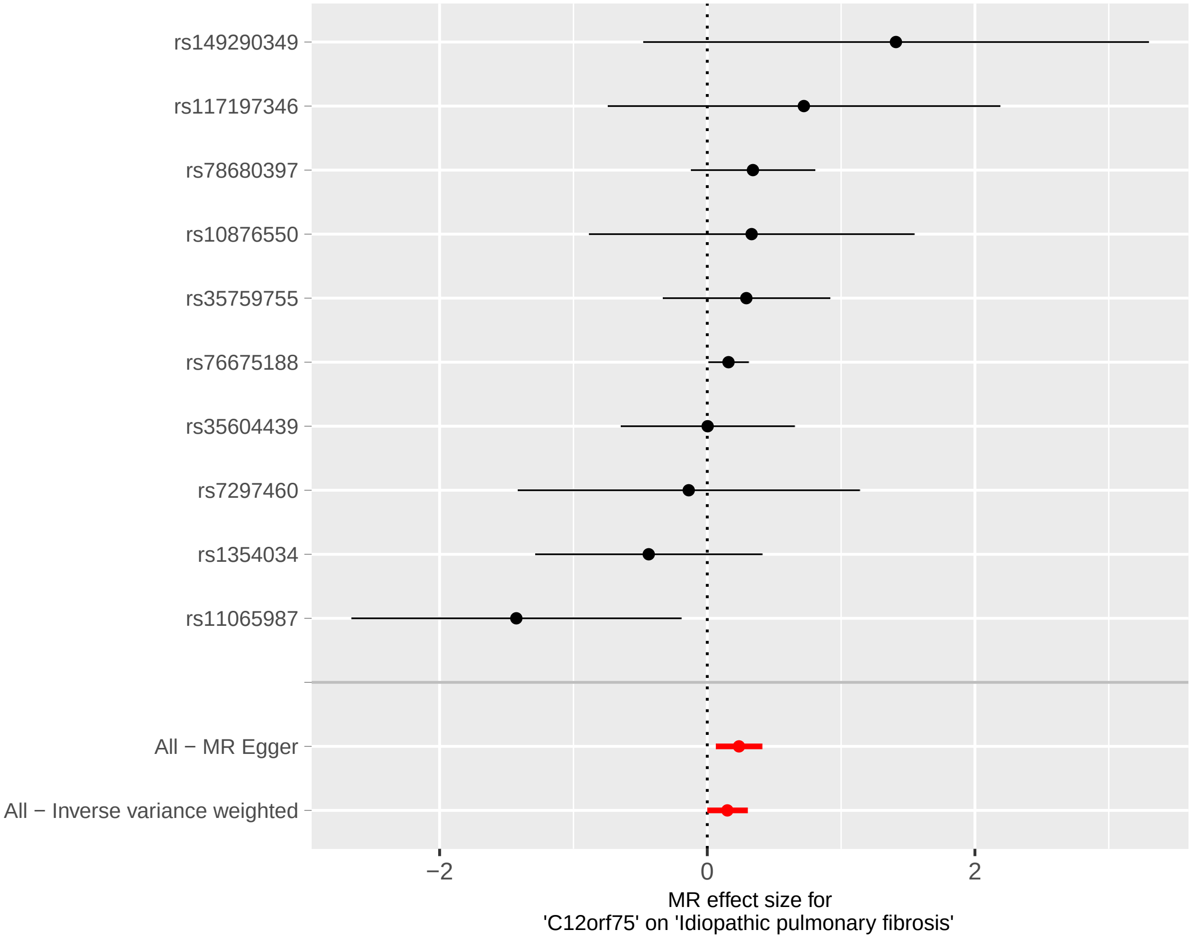
rs149290349
rs117197346
rs78680397
rs10876550
rs35759755
rs76675188
rs35604439
rs7297460
rs1354034
rs11065987
All − MR Egger
All − Inverse variance weighted
−2
0
2
MR effect size for
'C12orf75' on 'Idiopathic pulmonary fibrosis'

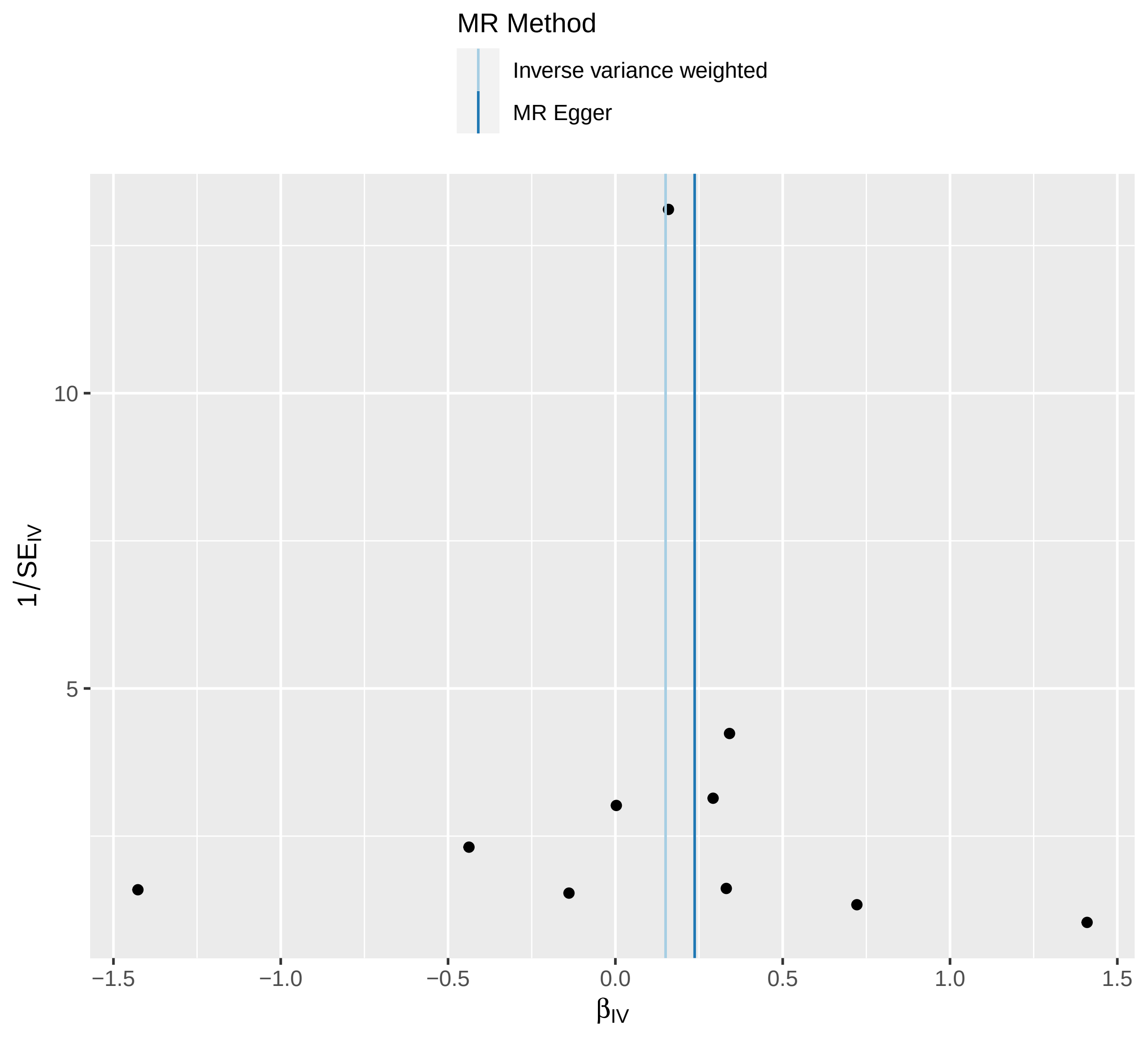
MR Method
Inverse variance weighted
MR Egger
10
5
$1/SE_{IV}$
−1.5
−1.0
−0.5
0.0
0.5
1.0
1.5
β_{IV}

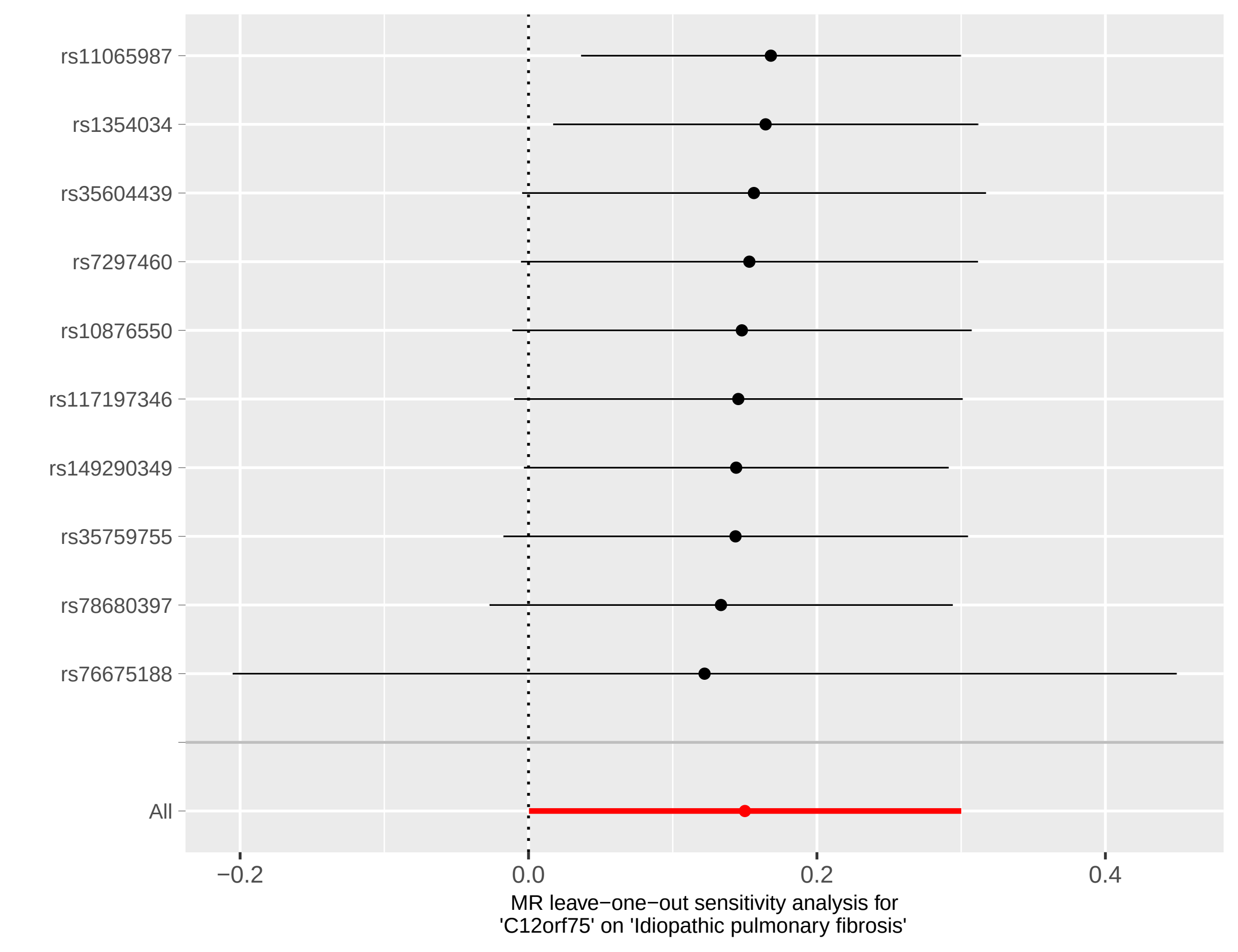
rs11065987
rs1354034
rs35604439
rs7297460
rs10876550
rs117197346
rs149290349
rs35759755
rs78680397
rs76675188
All
−0.2
0.0
0.2
0.4
MR leave−one−out sensitivity analysis for
'C12orf75' on 'Idiopathic pulmonary fibrosis'

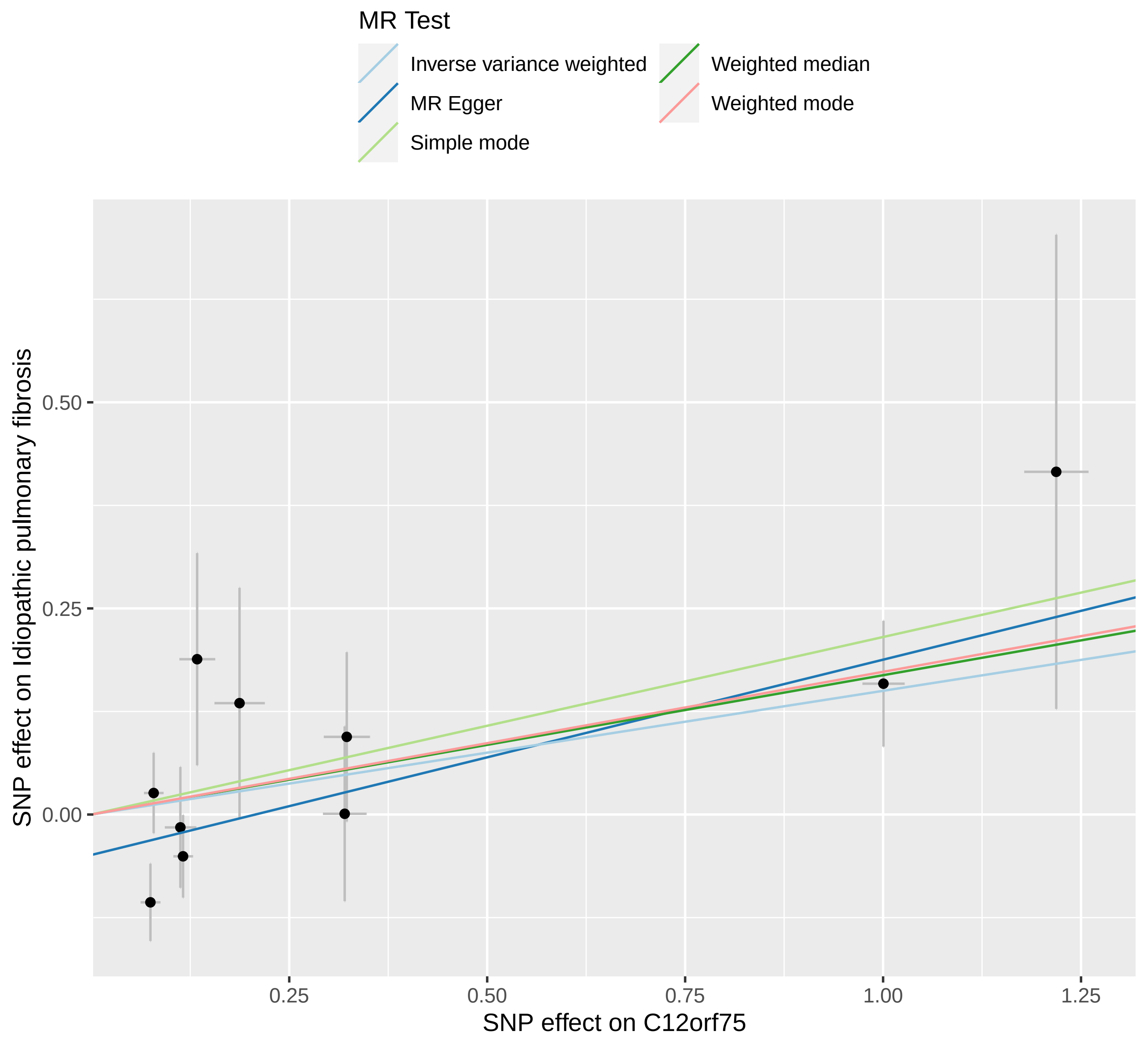
MR Test
Inverse variance weighted
MR Egger
Simple mode
Weighted median
Weighted mode
SNP effect on Idiopathic pulmonary fibrosis
0.50
0.25
0.00
0.25
0.50
0.75
1.00
1.25
SNP effect on C12orf75

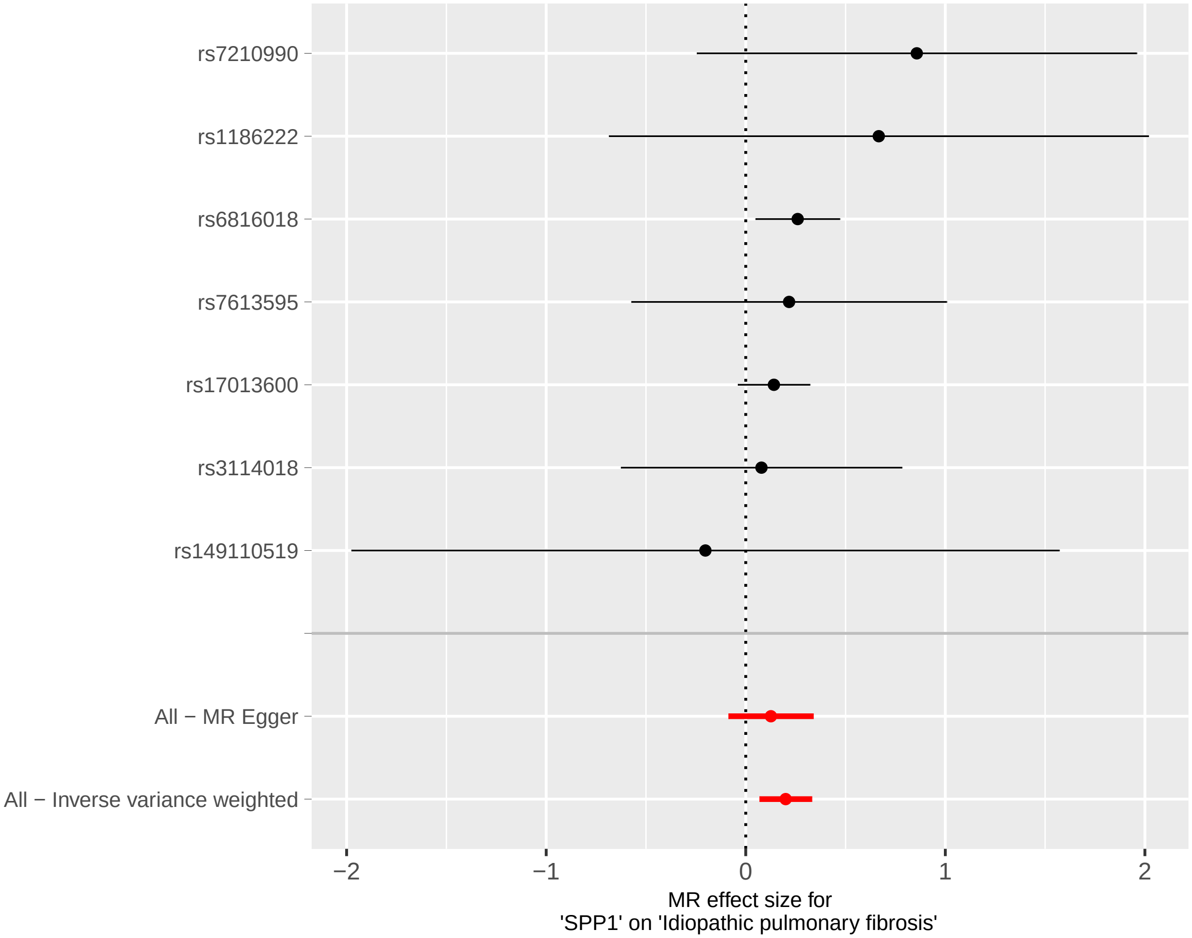
rs7210990
rs1186222
rs6816018
rs7613595
rs17013600
rs3114018
rs149110519
All − MR Egger
All − Inverse variance weighted
−2
−1
0
1
2
MR effect size for
'SPP1' on 'Idiopathic pulmonary fibrosis'

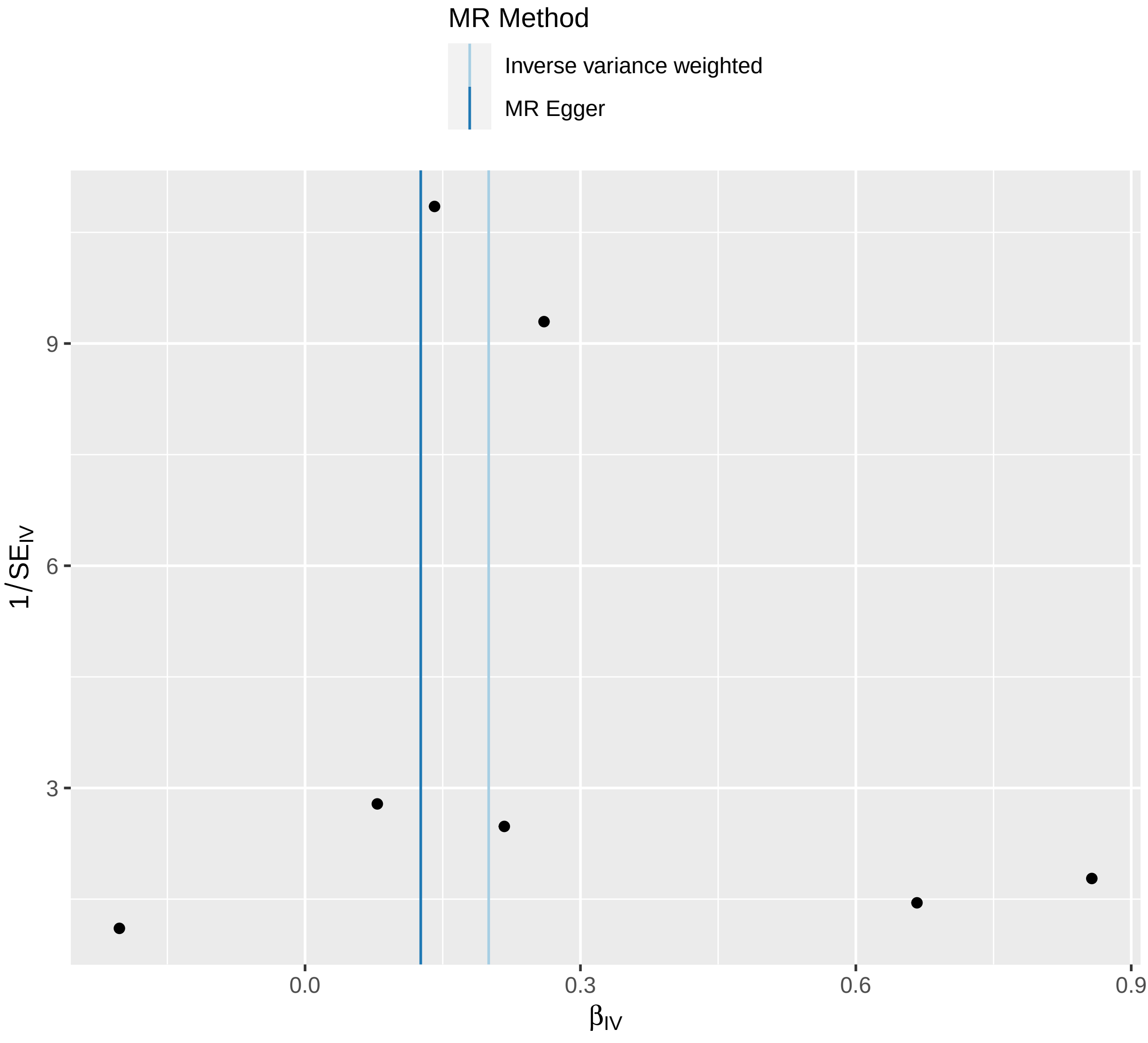

MR Method
Inverse variance weighted
MR Egger
1/SE_IV
9
6
3
0.0
0.3
0.6
0.9
β_IV

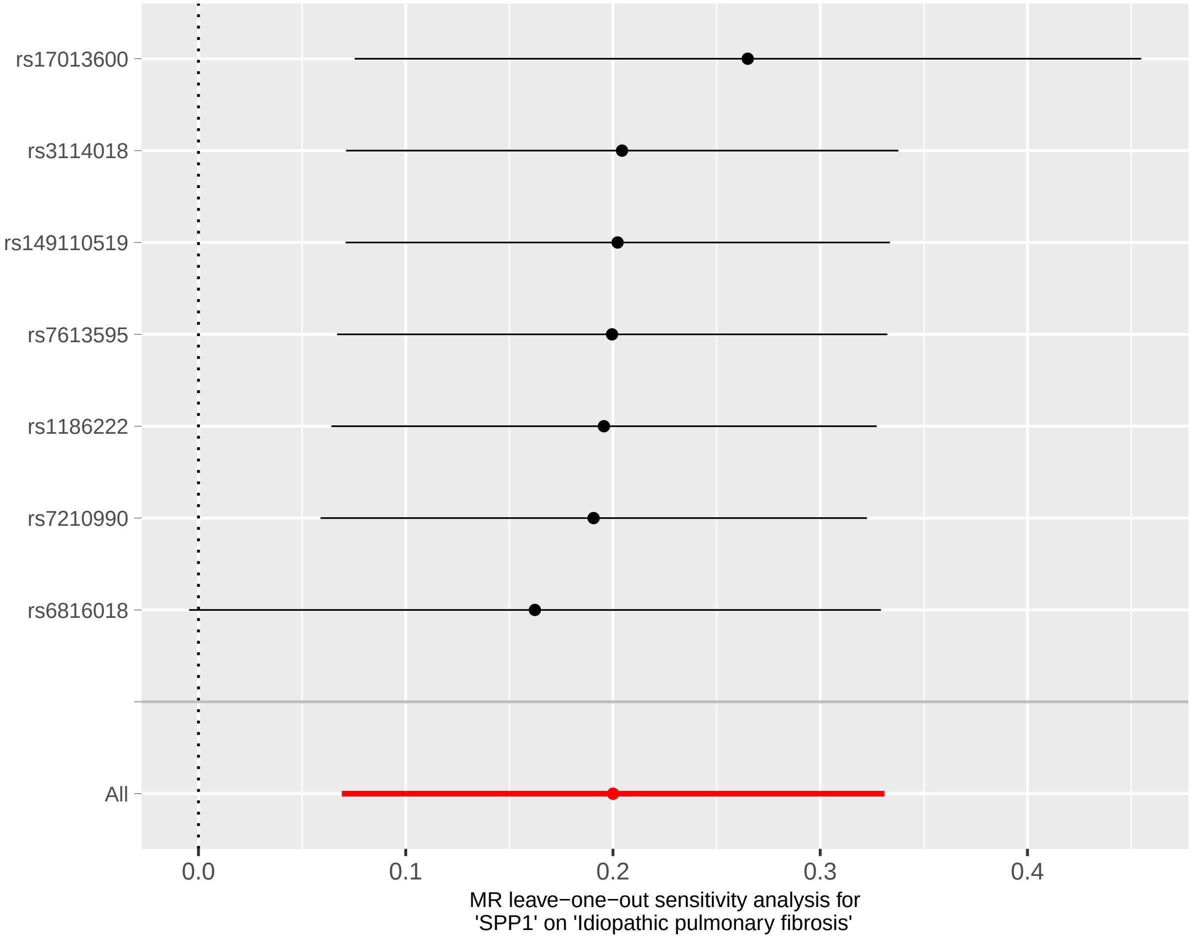
rs17013600
rs3114018
rs149110519
rs7613595
rs1186222
rs7210990
rs6816018
All
0.0
0.1
0.2
0.3
0.4
MR leave−one−out sensitivity analysis for
'SPP1' on 'Idiopathic pulmonary fibrosis'

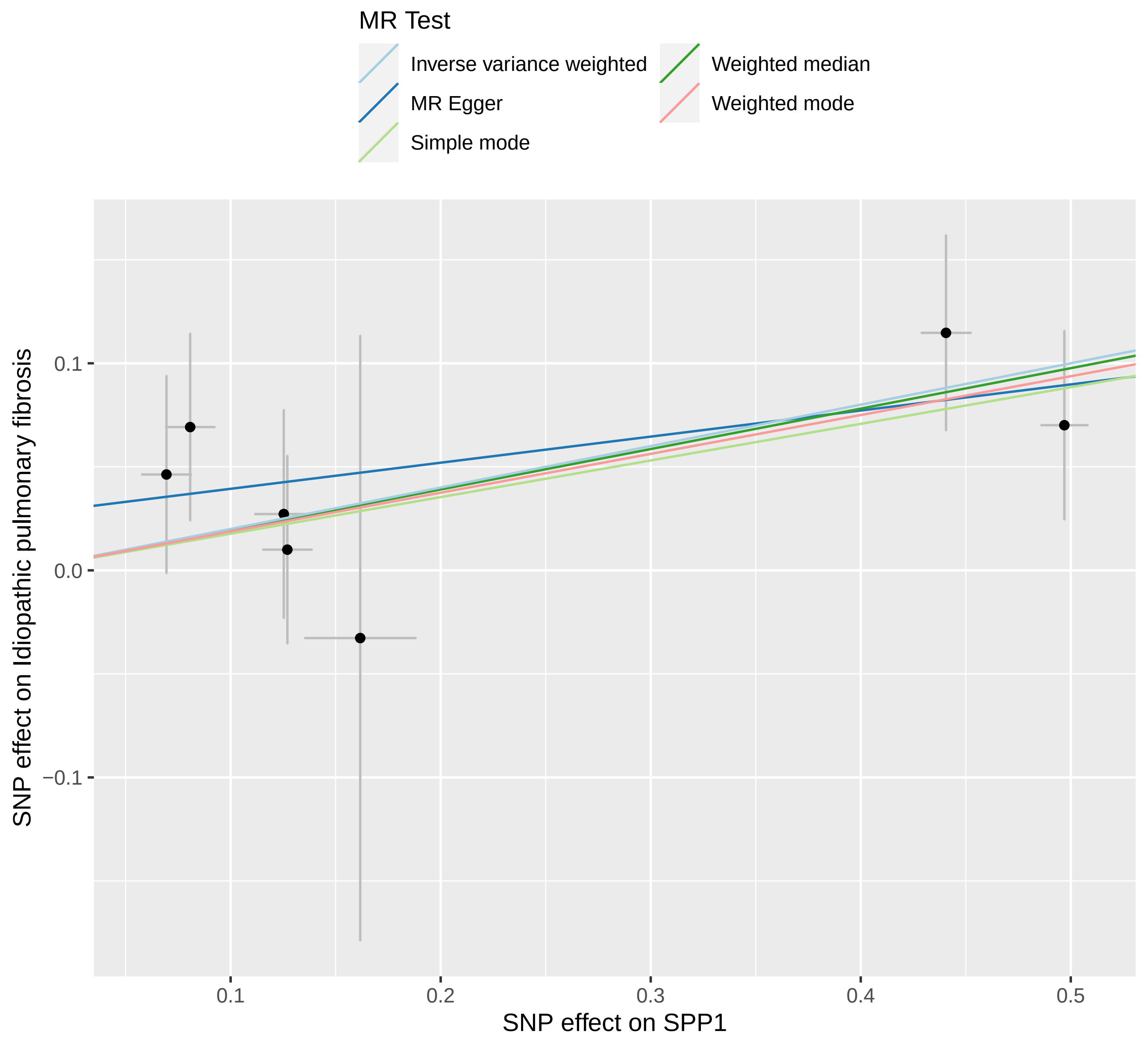

MR Test
Inverse variance weighted
MR Egger
Simple mode
Weighted median
Weighted mode
SNP effect on Idiopathic pulmonary fibrosis
0.1
0.0
−0.1
0.1
0.2
0.3
0.4
0.5
SNP effect on SPP1

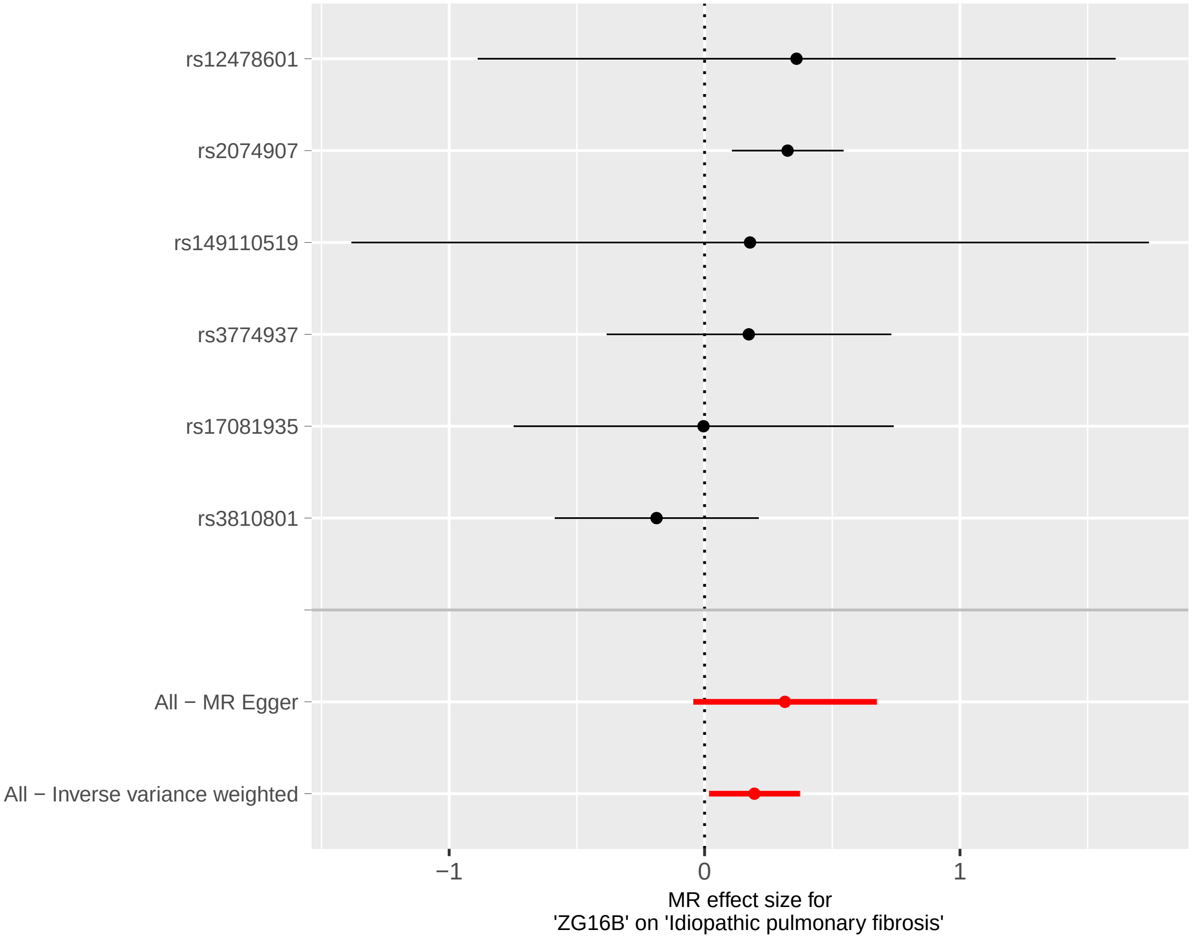
rs12478601
rs2074907
rs149110519
rs3774937
rs17081935
rs3810801
All − MR Egger
All − Inverse variance weighted
−1
0
1
MR effect size for
'ZG16B' on 'Idiopathic pulmonary fibrosis'

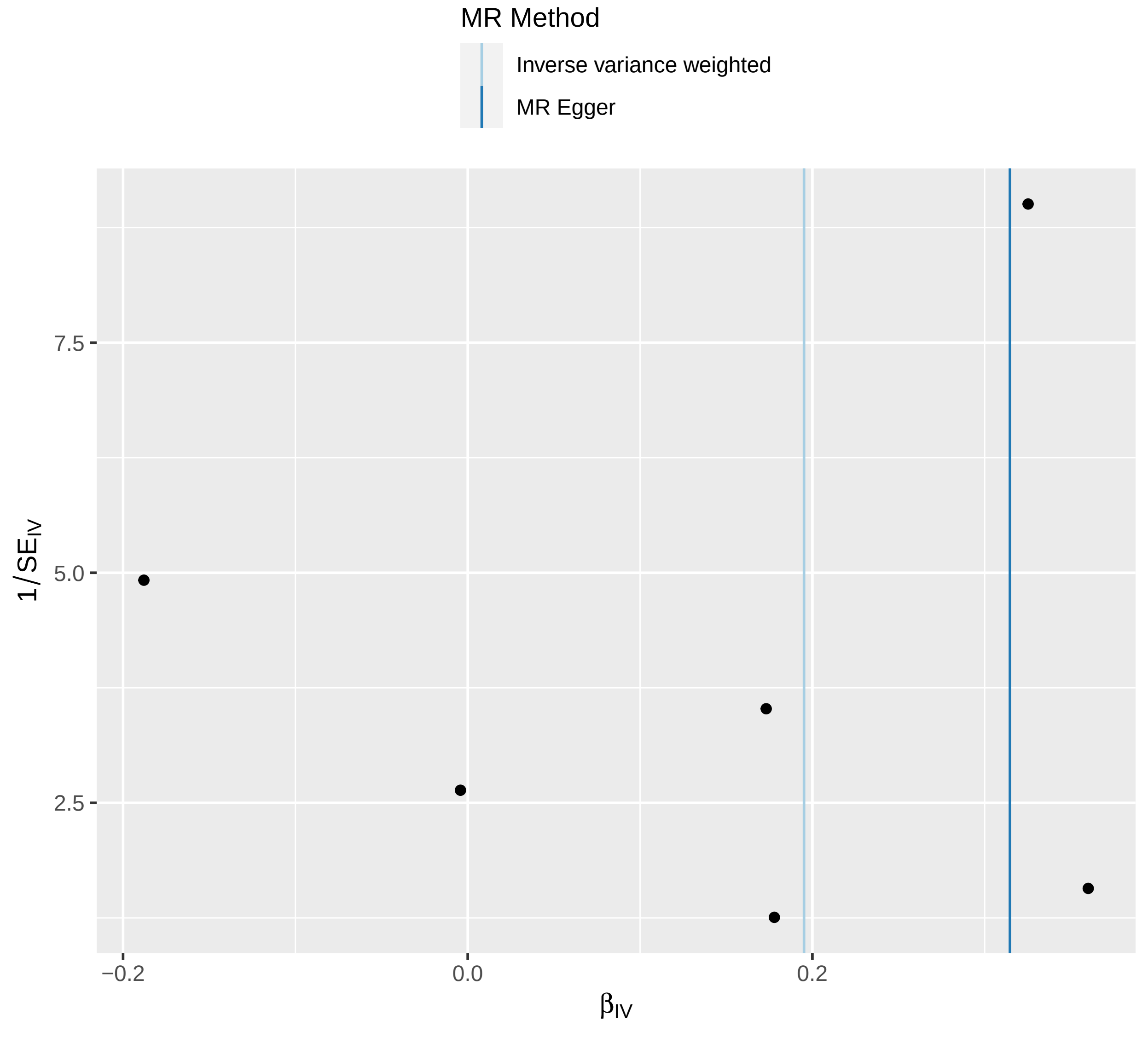
MR Method
Inverse variance weighted
MR Egger
7.5
5.0
2.5
1/SE$_{IV}$
−0.2
0.0
0.2
β$_{IV}$

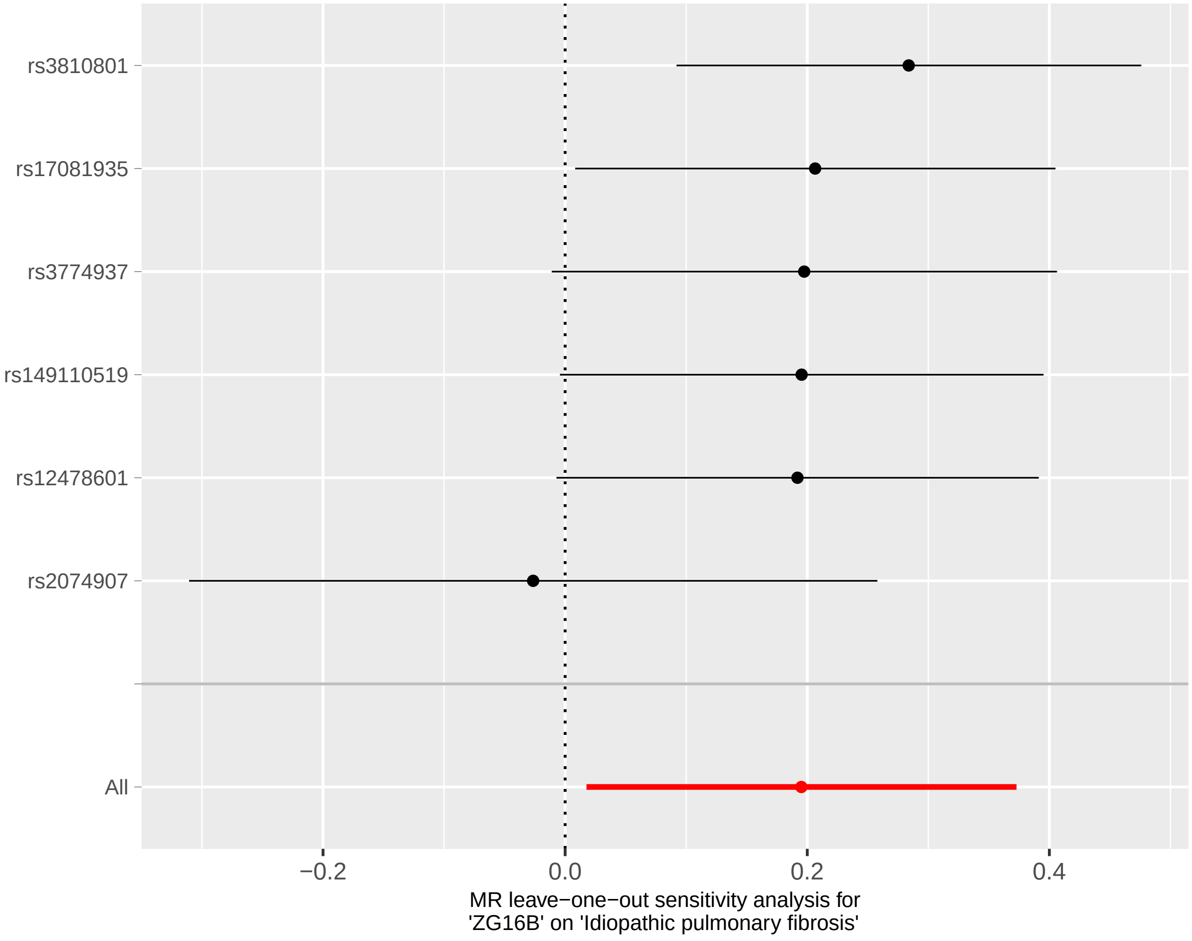
rs3810801
rs17081935
rs3774937
rs149110519
rs12478601
rs2074907
All
−0.2
0.0
0.2
0.4
MR leave−one−out sensitivity analysis for
'ZG16B' on 'Idiopathic pulmonary fibrosis'

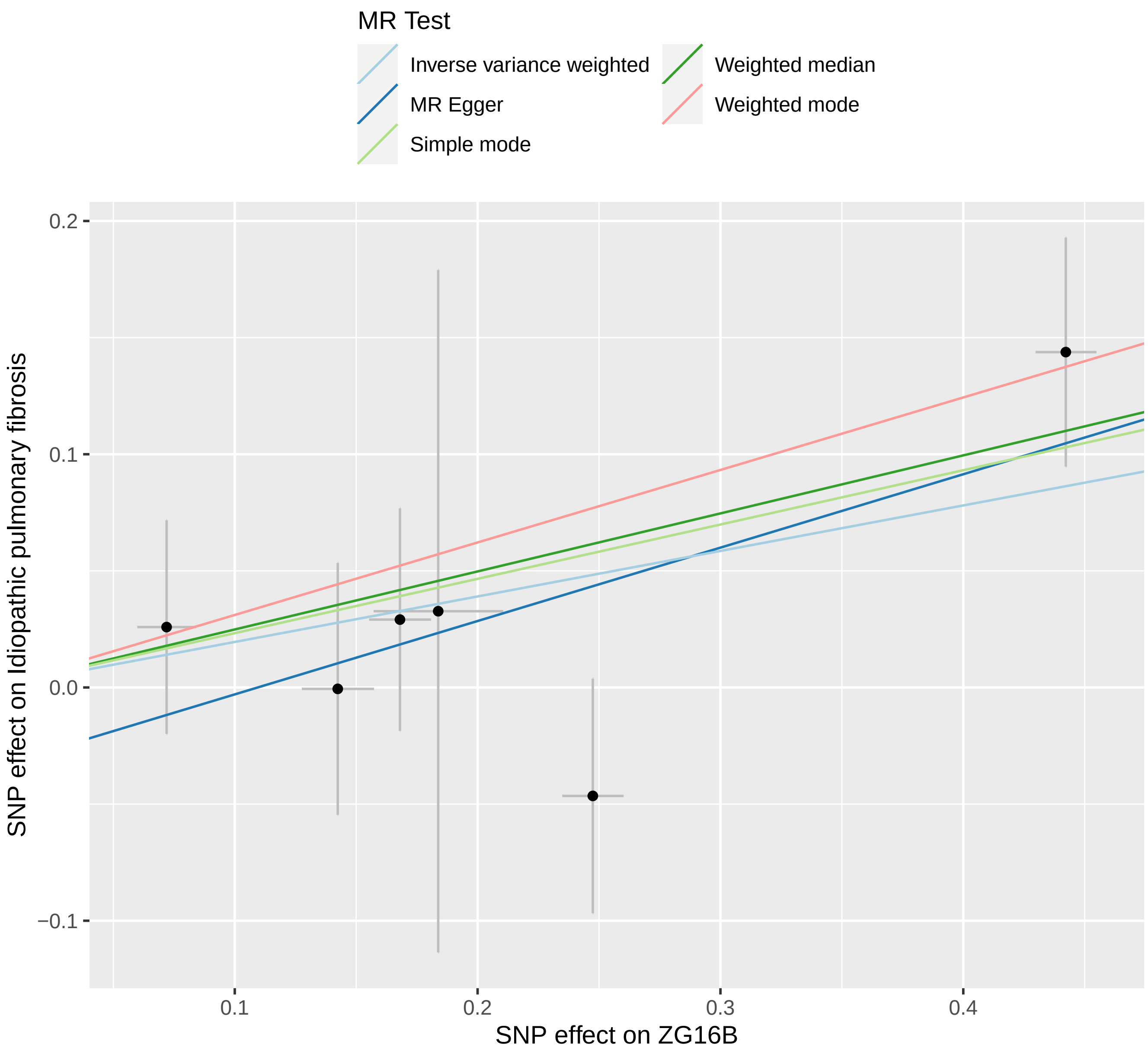

MR Test
Inverse variance weighted
MR Egger
Simple mode
Weighted median
Weighted mode
0.2
0.1
0.0
−0.1
0.1
0.2
0.3
0.4
SNP effect on Idiopathic pulmonary fibrosis
SNP effect on ZG16B

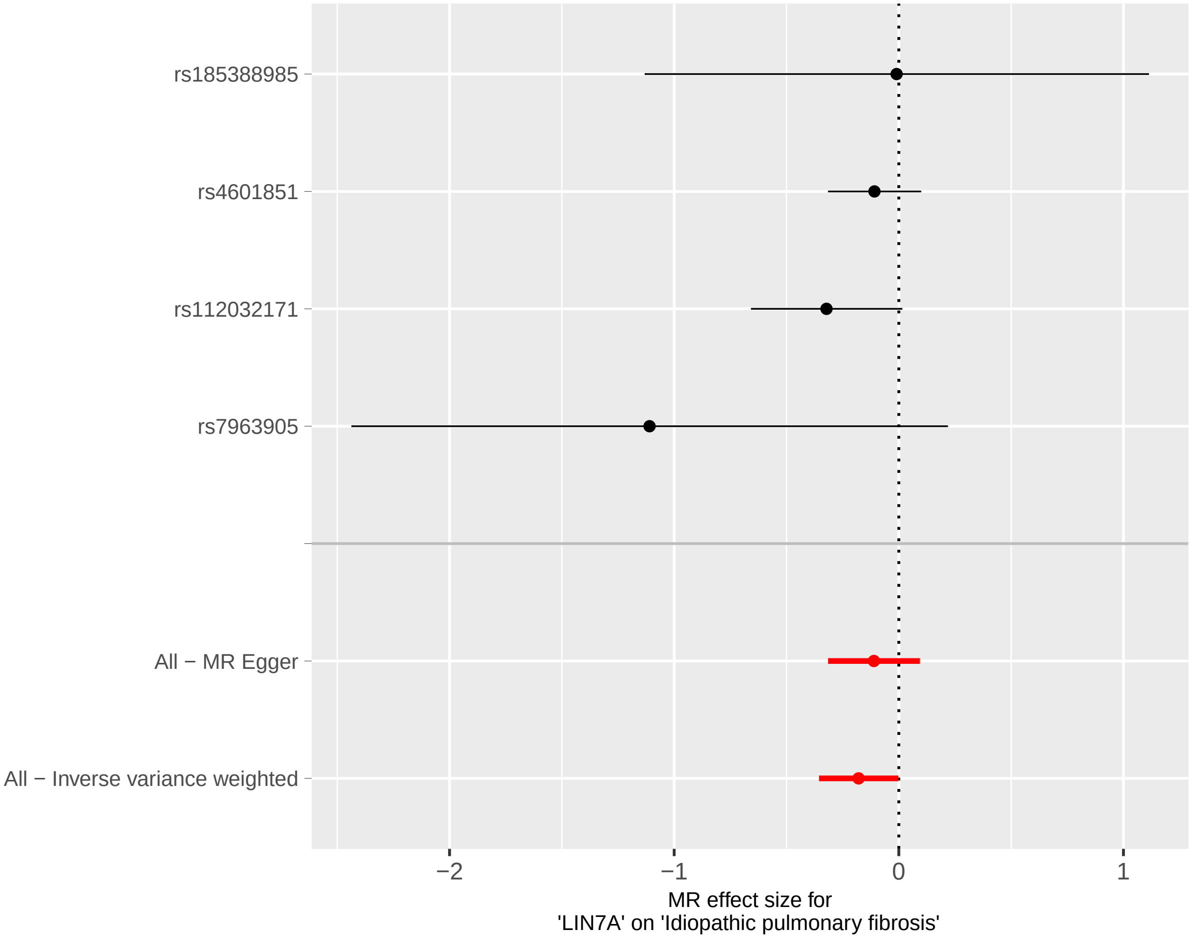
rs185388985
rs4601851
rs112032171
rs7963905
All − MR Egger
All − Inverse variance weighted
−2
−1
0
1
MR effect size for
'LIN7A' on 'Idiopathic pulmonary fibrosis'

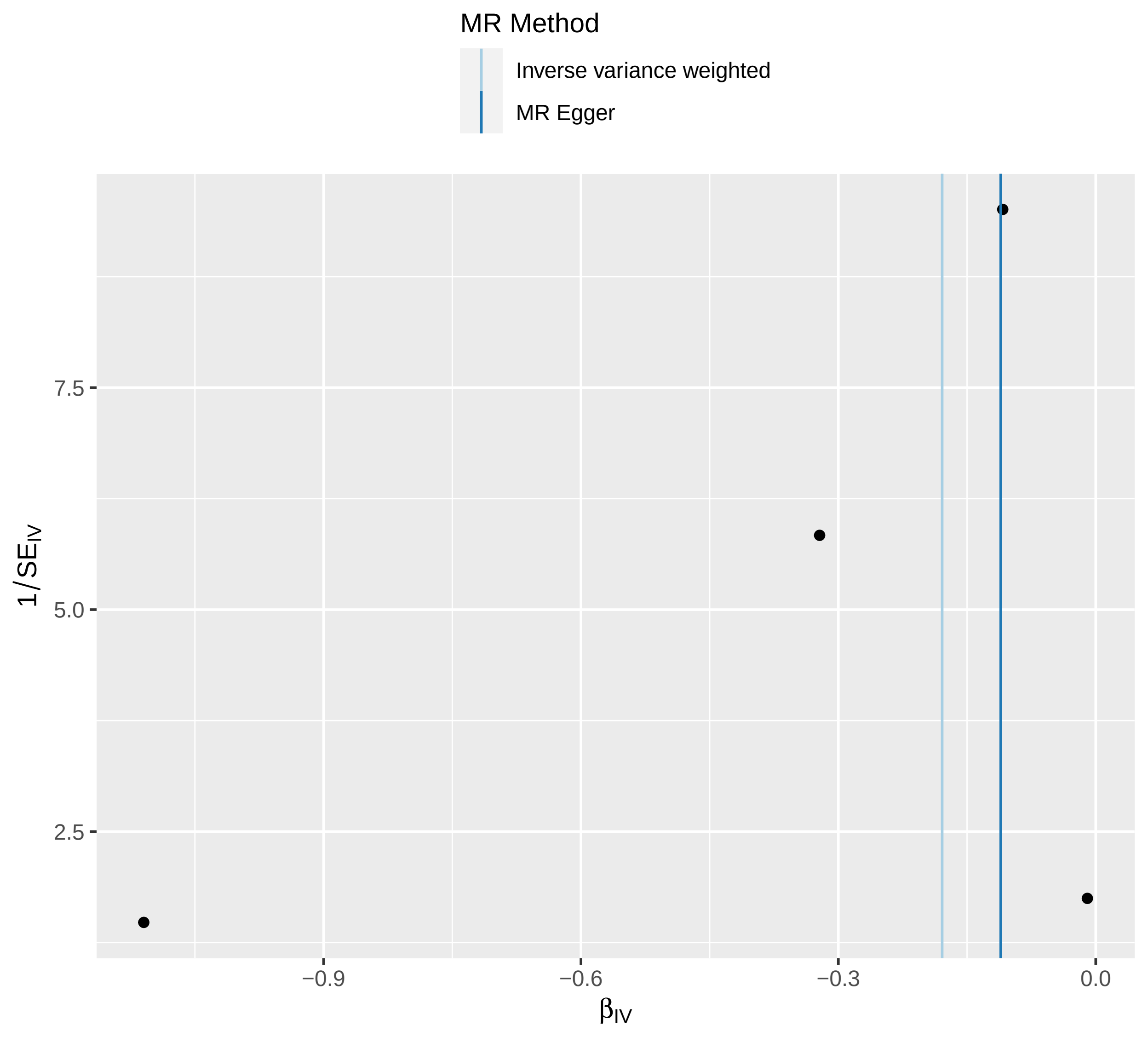
MR Method
Inverse variance weighted
MR Egger
7.5
5.0
2.5
$1/SE_{IV}$
−0.9
−0.6
−0.3
0.0
β_{IV}

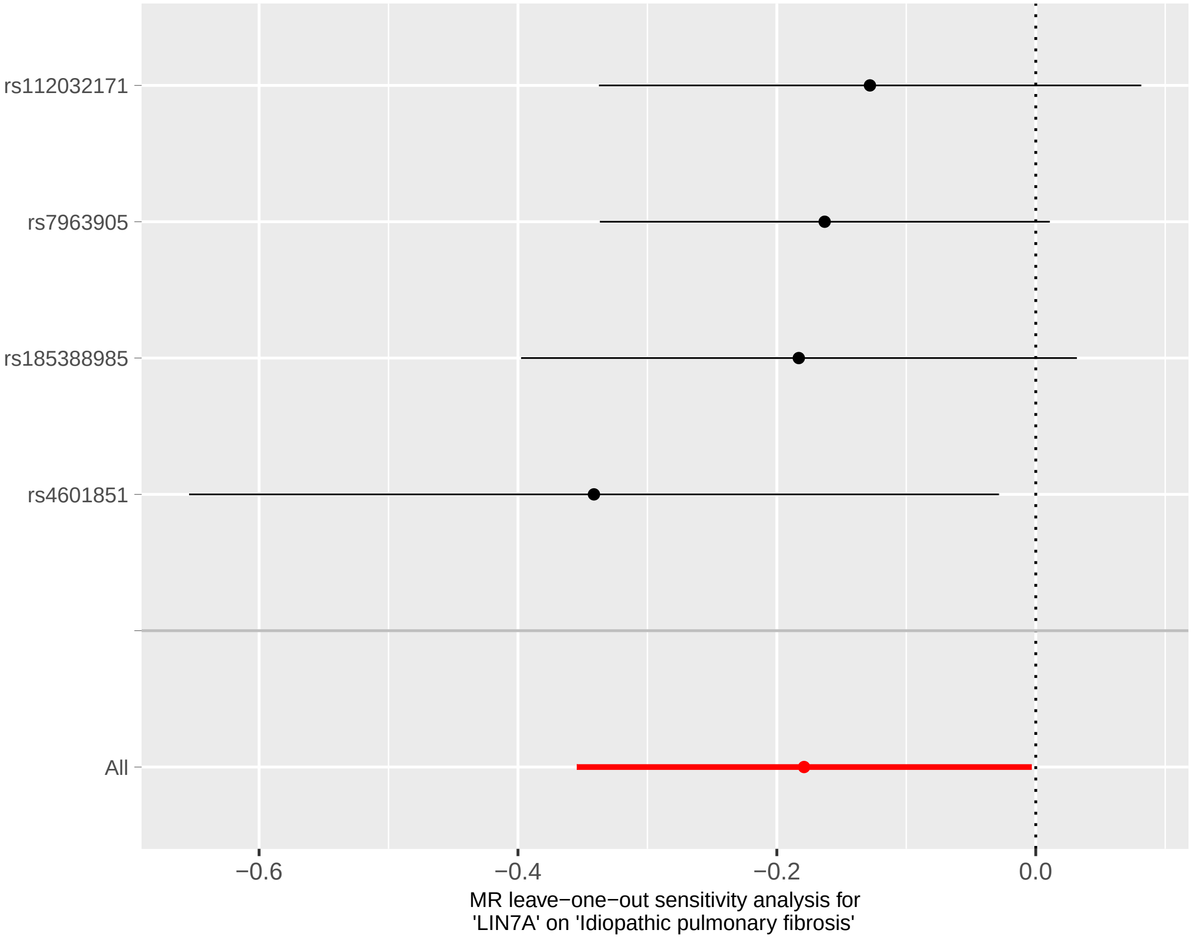
rs112032171
rs7963905
rs185388985
rs4601851
All
−0.6
−0.4
−0.2
0.0
MR leave−one−out sensitivity analysis for
'LIN7A' on 'Idiopathic pulmonary fibrosis'

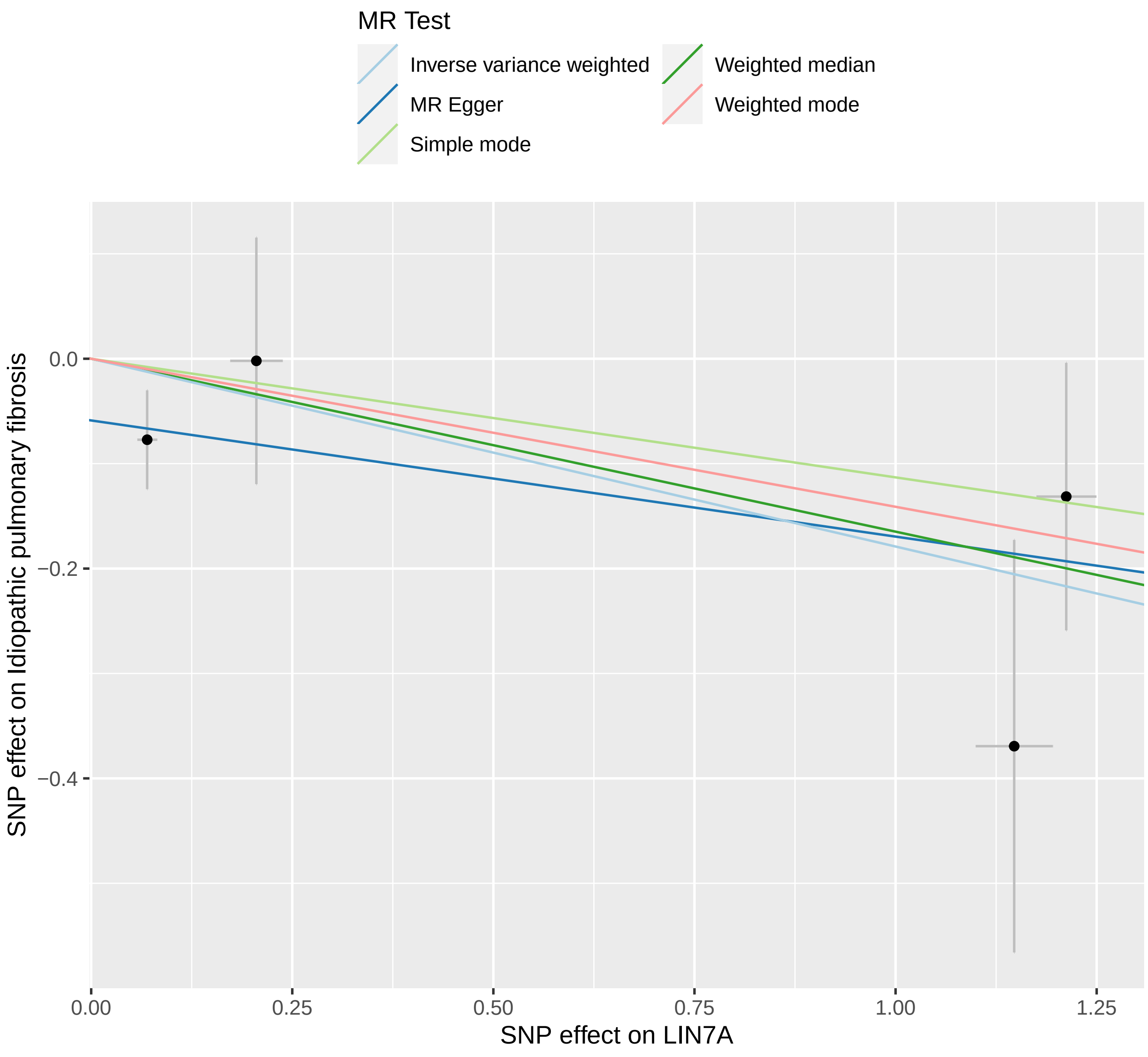
MR Test
Inverse variance weighted
MR Egger
Simple mode
Weighted median
Weighted mode
SNP effect on Idiopathic pulmonary fibrosis
0.0
−0.2
−0.4
0.00
0.25
0.50
0.75
1.00
1.25
SNP effect on LIN7A

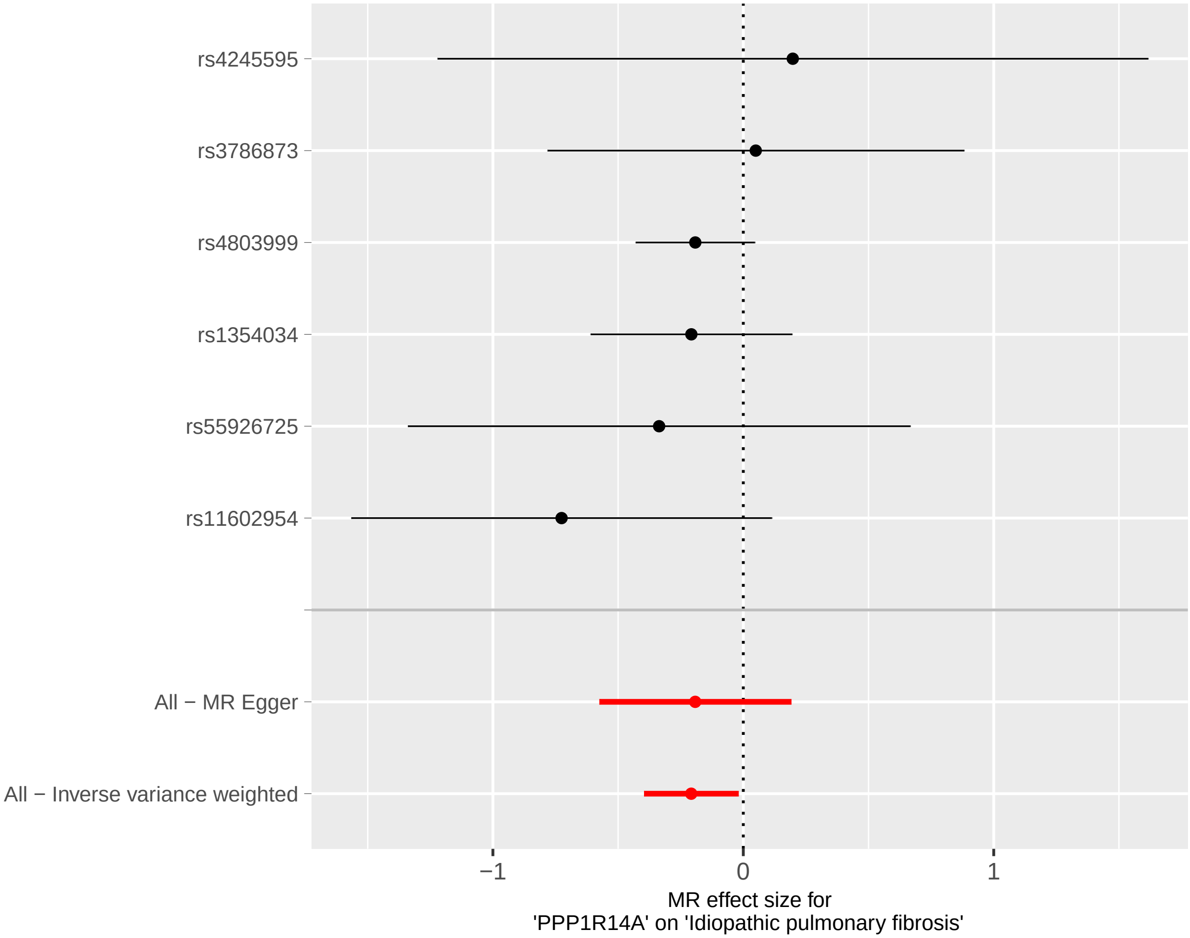

rs4245595
rs3786873
rs4803999
rs1354034
rs55926725
rs11602954
All − MR Egger
All − Inverse variance weighted
−1
0
1
MR effect size for
'PPP1R14A' on 'Idiopathic pulmonary fibrosis'

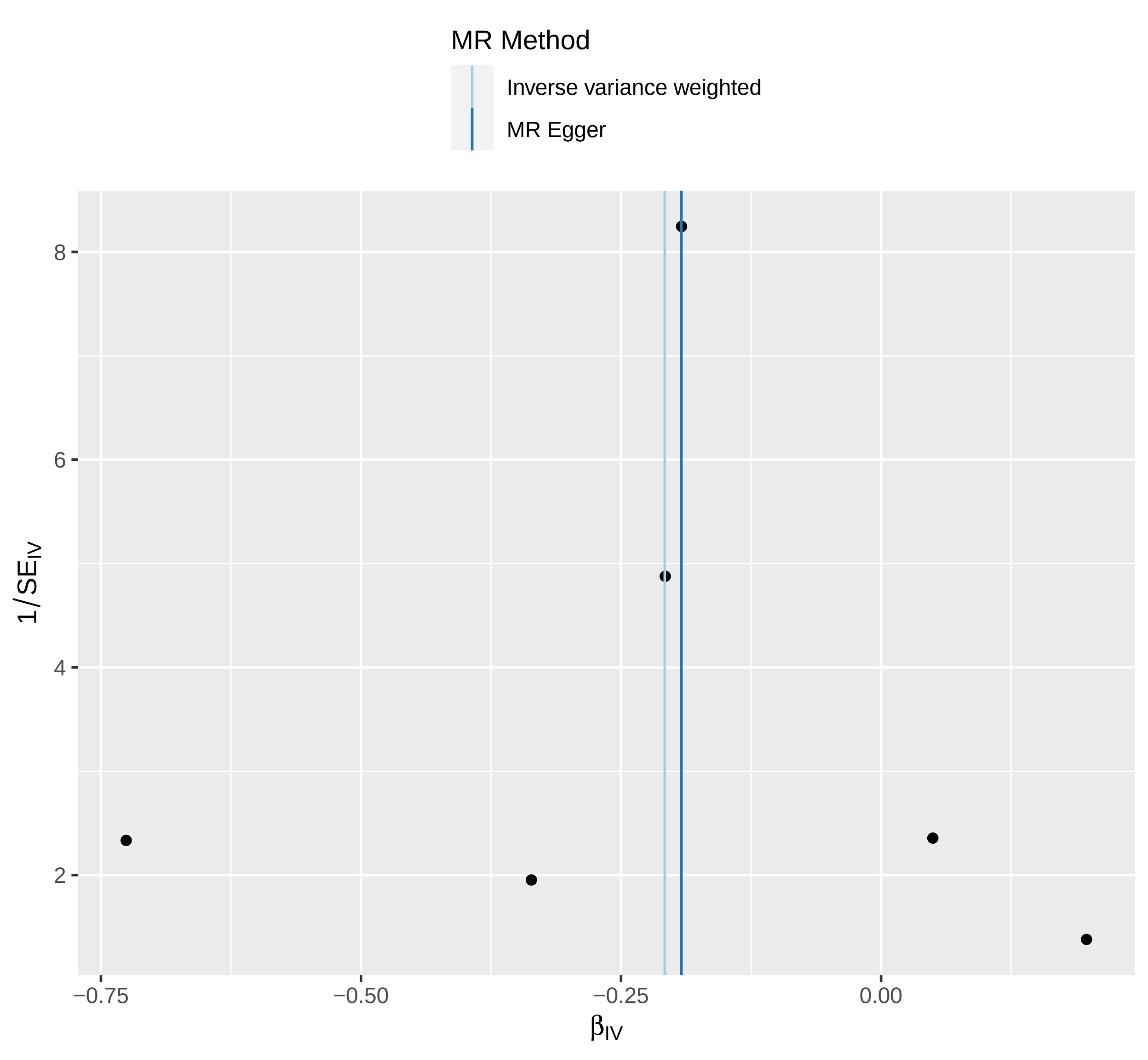
MR Method
Inverse variance weighted
MR Egger
8
6
4
2
1/SE_IV
−0.75
−0.50
−0.25
0.00
β_IV

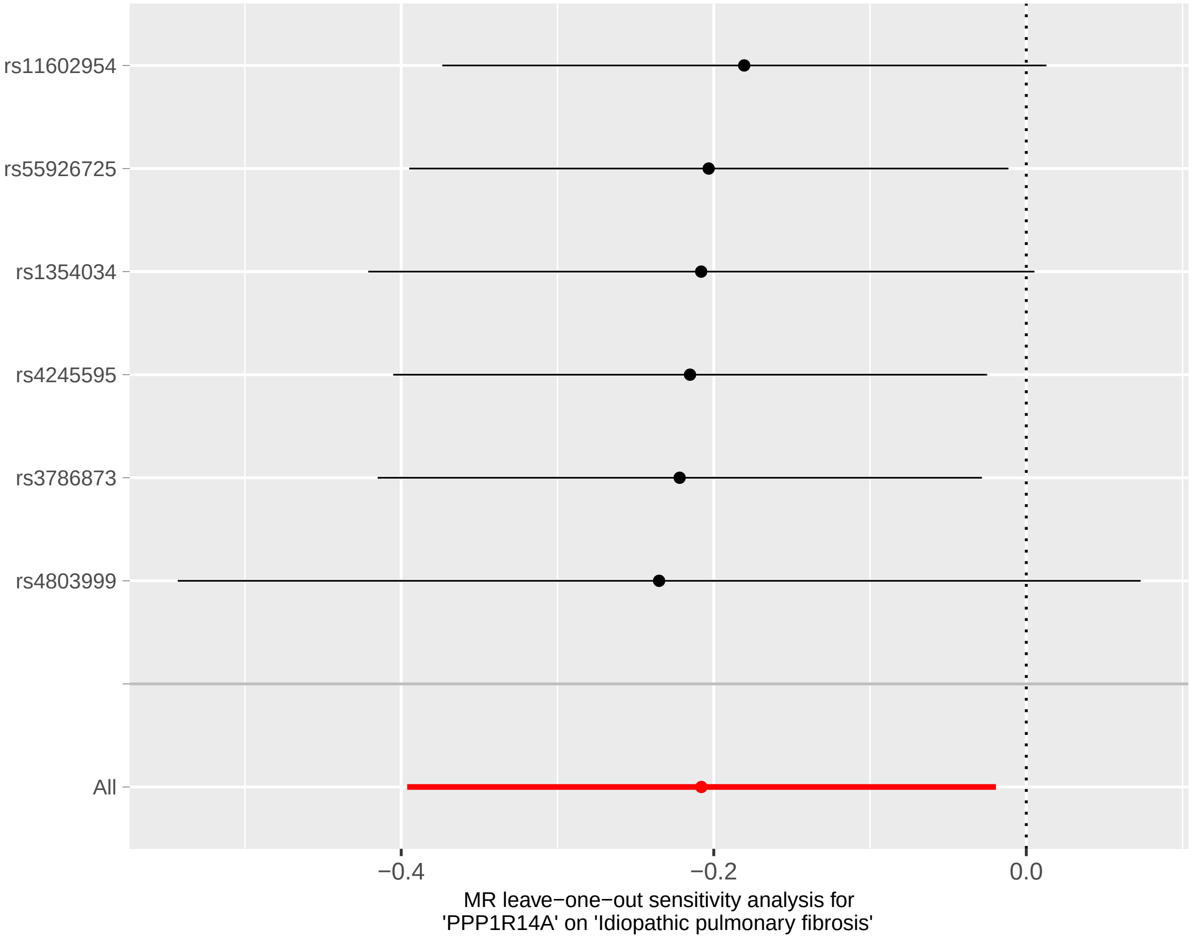
rs11602954
rs55926725
rs1354034
rs4245595
rs3786873
rs4803999
All
−0.4
−0.2
0.0
MR leave−one−out sensitivity analysis for
'PPP1R14A' on 'Idiopathic pulmonary fibrosis'

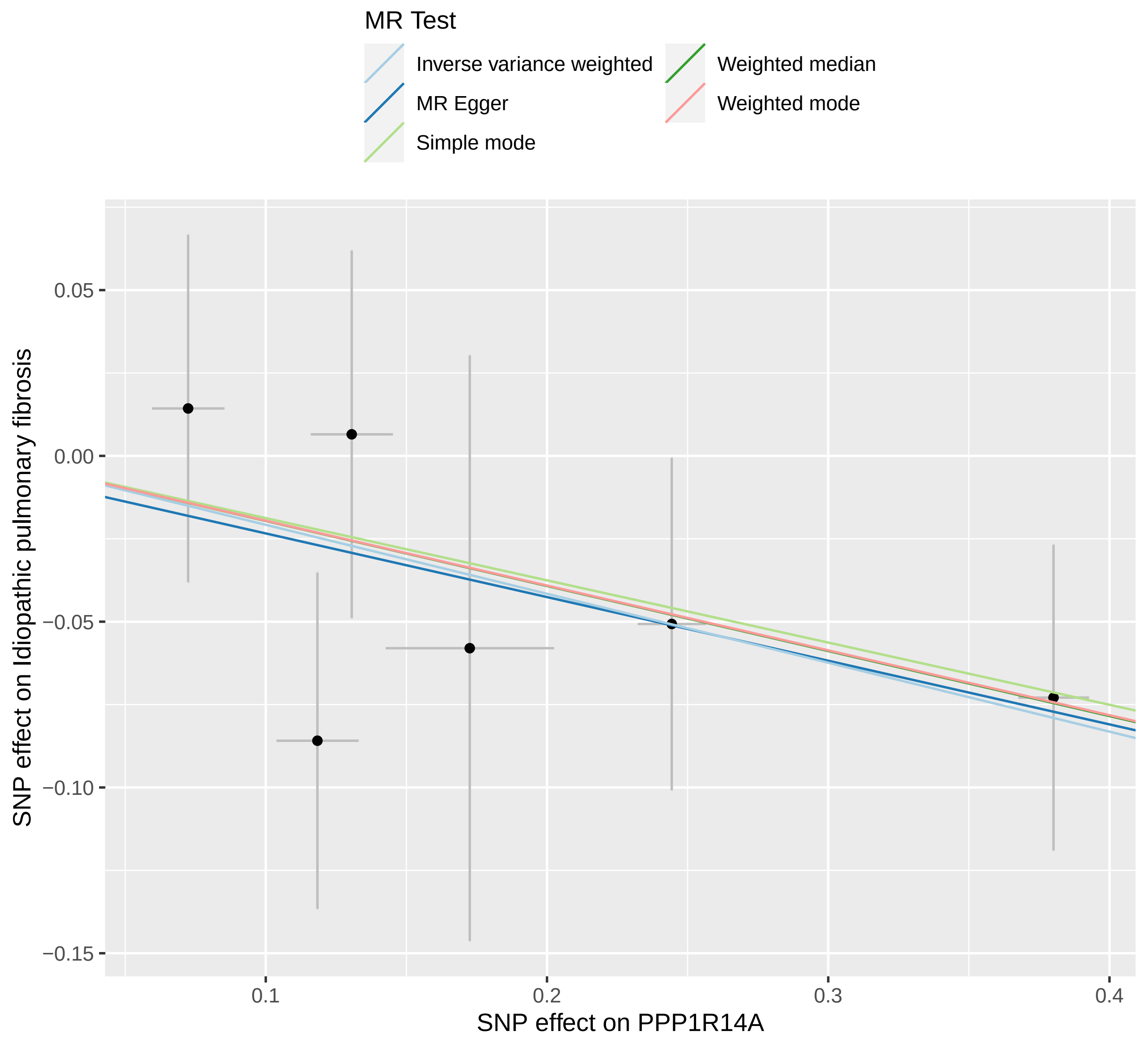
MR Test
Inverse variance weighted
MR Egger
Simple mode
Weighted median
Weighted mode
0.05
0.00
−0.05
−0.10
−0.15
0.1
0.2
0.3
0.4
SNP effect on Idiopathic pulmonary fibrosis
SNP effect on PPP1R14A

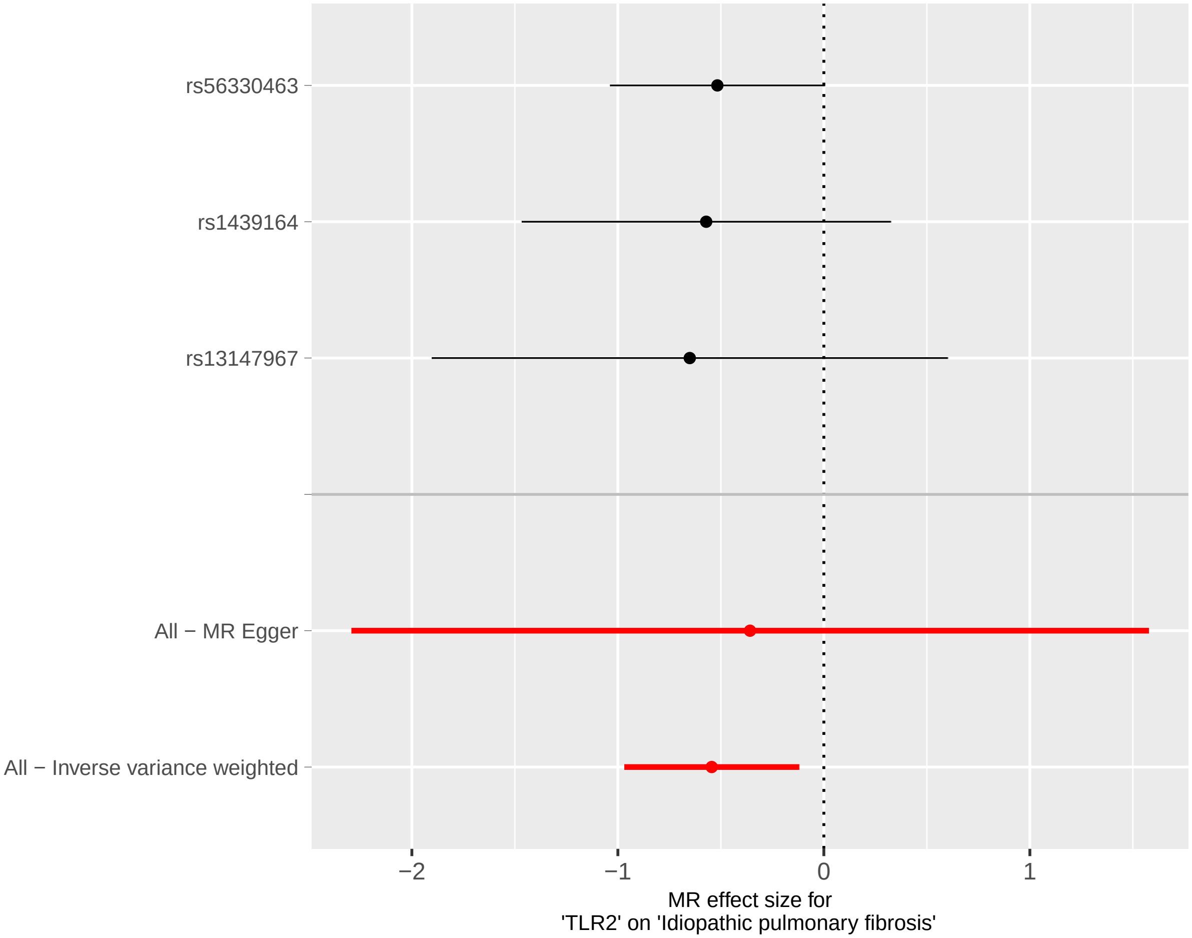

rs56330463
rs1439164
rs13147967
All − MR Egger
All − Inverse variance weighted
−2
−1
0
1
MR effect size for
'TLR2' on 'Idiopathic pulmonary fibrosis'

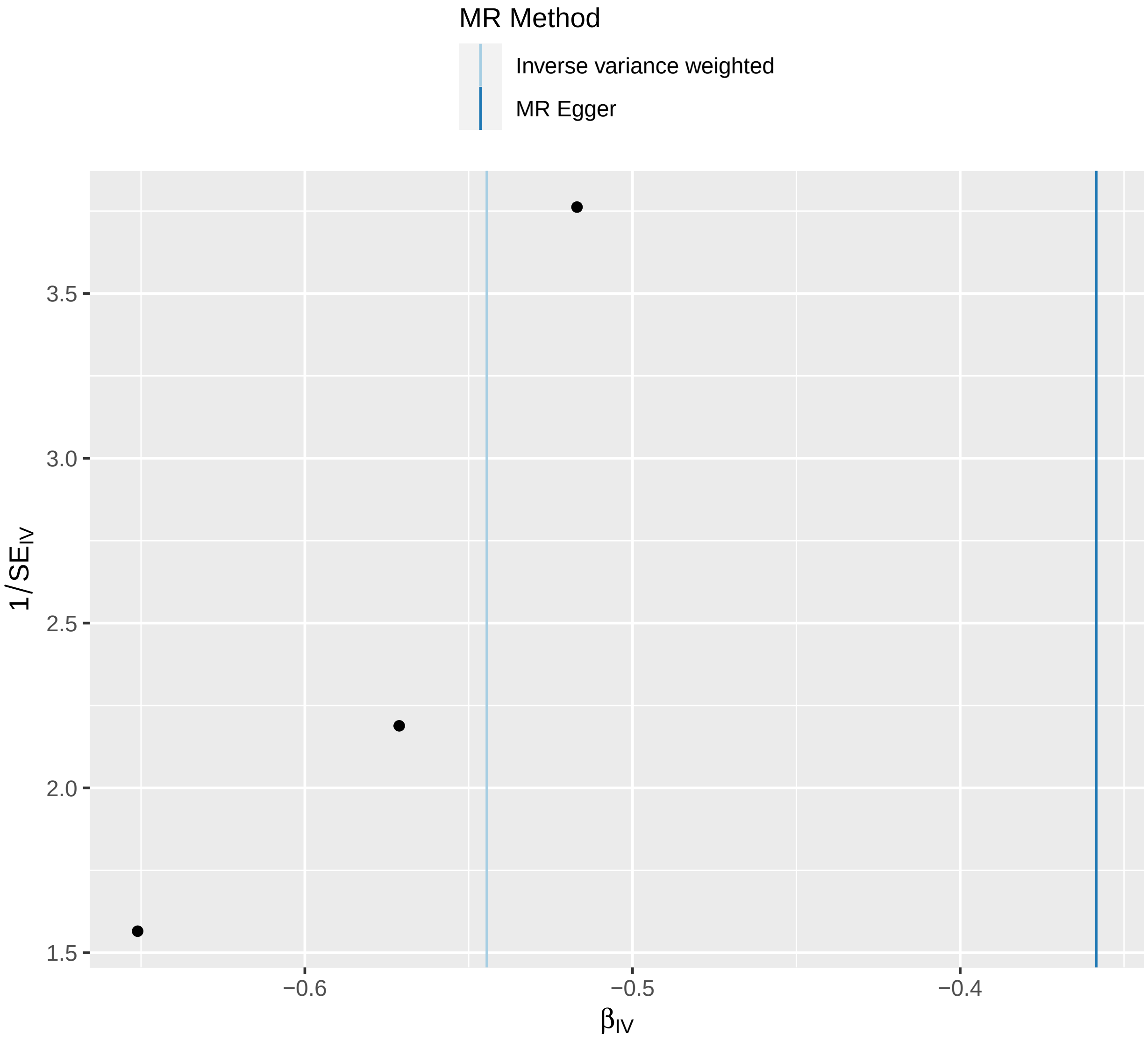
MR Method
Inverse variance weighted
MR Egger
3.5
3.0
2.5
2.0
1.5
1/SE$_{IV}$
−0.6
−0.5
−0.4
β$_{IV}$

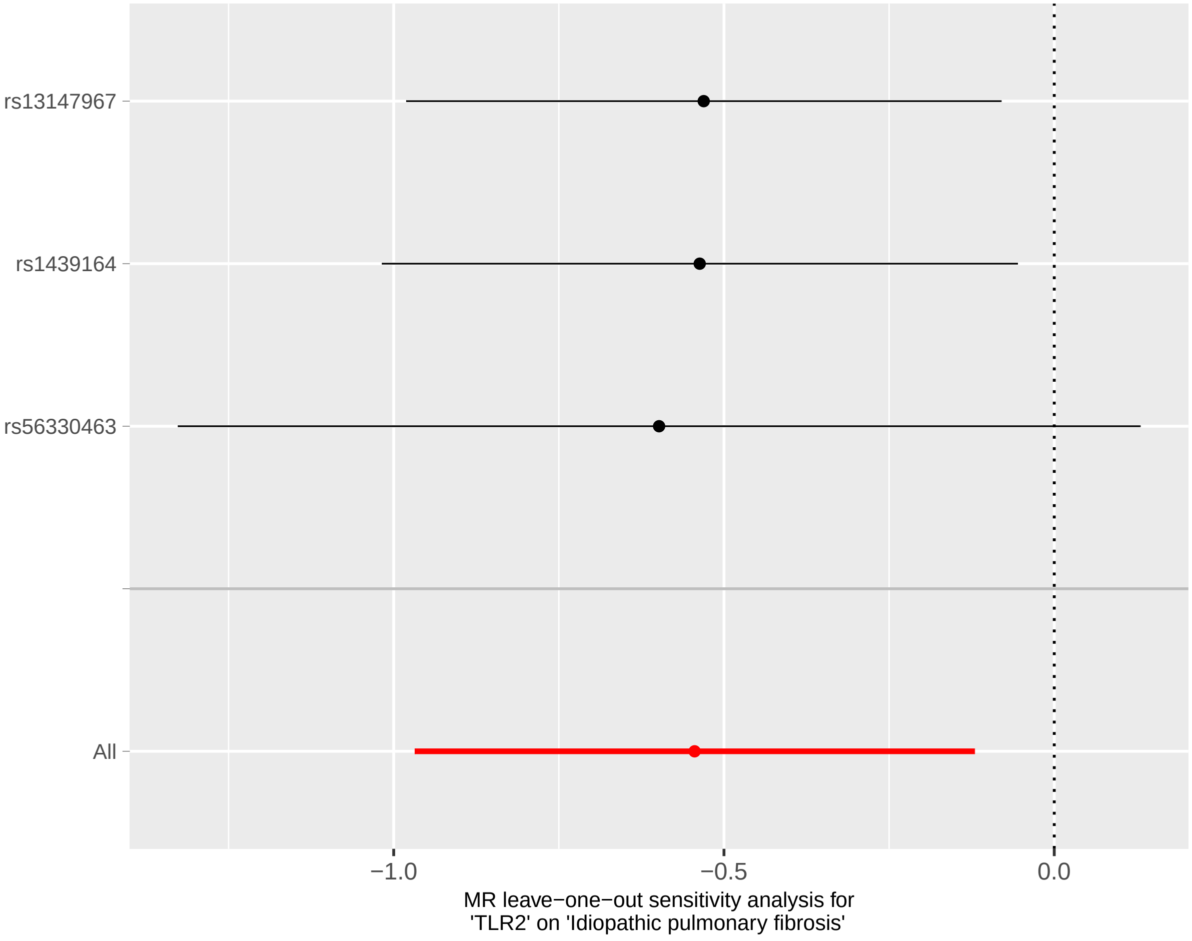

rs13147967
rs1439164
rs56330463
All
−1.0
−0.5
0.0
MR leave−one−out sensitivity analysis for
'TLR2' on 'Idiopathic pulmonary fibrosis'

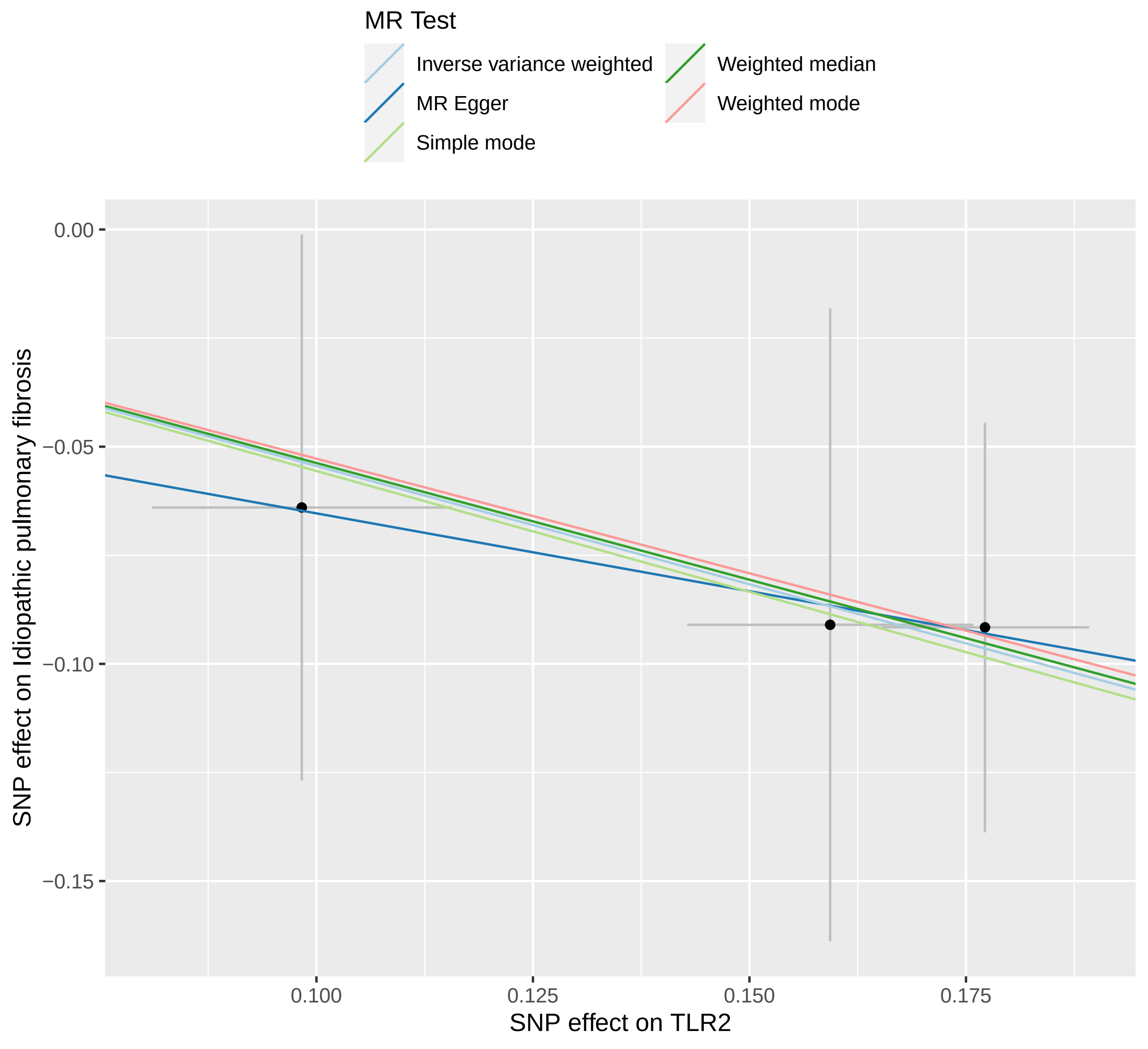
MR Test
Inverse variance weighted
MR Egger
Simple mode
Weighted median
Weighted mode
0.00
−0.05
−0.10
−0.15
SNP effect on Idiopathic pulmonary fibrosis
0.100
0.125
0.150
0.175
SNP effect on TLR2
